# A robust yeast chassis: comprehensive characterization of a fast-growing *Saccharomyces cerevisiae*

**DOI:** 10.1128/mbio.03196-23

**Published:** 2024-01-12

**Authors:** Yangdanyu Long, Xiao Han, Xuanlin Meng, Ping Xu, Fei Tao

**Affiliations:** 1 State Key Laboratory of Microbial Metabolism and School of Life Sciences & Biotechnology, Shanghai Jiao Tong University, Shanghai, China; Korea Advanced Institute of Science and Technology, Daejon, South Korea

**Keywords:** *Saccharomyces cerevisiae*, growth rate, synthetic biology, chassis, l-lactic acid, robustness

## Abstract

Robust chassis are critical to facilitate advances in synthetic biology. This study describes a comprehensive characterization of a new yeast isolate *Saccharomyces cerevisiae* XP that grows faster than commonly used research and industrial *S. cerevisiae* strains. The genomic, transcriptomic, and metabolomic analyses suggest that the fast growth rate is, in part, due to the efficient electron transport chain and key growth factor synthesis. A toolbox for genetic manipulation of the yeast was developed; we used it to construct l-lactic acid producers for high lactate production. The development of genetically malleable yeast strains that grow faster than currently used strains may significantly enhance the uses of *S. cerevisiae* in biotechnology.

**IMPORTANCE:**

Yeast is known as an outstanding starting strain for constructing microbial cell factories. However, its growth rate restricts its application. A yeast strain XP, which grows fast in high concentrations of sugar and acidic environments, is revealed to demonstrate the potential in industrial applications. A toolbox was also built for its genetic manipulation including gene insertion, deletion, and ploidy transformation. The knowledge of its metabolism, which could guide the designing of genetic experiments, was generated with multi-omics analyses. This novel strain along with its toolbox was then tested by constructing an l-lactic acid efficient producer, which is conducive to the development of degradable plastics. This study highlights the remarkable competence of nonconventional yeast for applications in biotechnology.

## INTRODUCTION

*Saccharomyces cerevisiae* is useful as a common chassis for synthetic biology because of its generally recognized as safe (GRAS) status, compatibility with industrial fermentation, and high tolerance to final products (1, 2). Currently, ethanol production by *S. cerevisiae* has reached the industrial scale.

*S. cerevisiae* is an irreplaceable chassis for industrial applications for several reasons. First, post-translational modifications and organelle systems in *S. cerevisiae* are conducive to the localization and expression of enzymes (3). Most eukaryotic cytochrome P450 enzymes (P450s) are endoplasmic reticulum localized membrane proteins (3). For example, the membrane-bound eukaryotic cytochrome P450 gene *cyp5150l8* was only successfully expressed in *S. cerevisiae* and produced 14.5 mg/L antitumor ganoderic acids (4). Second, *S. cerevisiae* is better suited to express genes from plants and animals because it is more closely related to them (5). The mevalonate pathway in *S. cerevisiae* is identical to that in plants (6). Artemisinic acid, the precursor of the antimalarial artemisinin, was efficiently synthesized in *S. cerevisiae* at a titer of 25.0 g/L (7). While it was synthesized in *E. coli* with a less titer of 105 (±10) mg/L (8). Downstream products in the mevalonate pathway, including sterols (9) and terpenoids (9–11), have been successfully produced in *S. cerevisiae*; however, only a handful of these can be efficiently produced in prokaryotes. Because of these advantages, *S. cerevisiae* has been used to produce unique high-value natural compounds (12). *De novo* synthesis of the opioid compounds thebaine and hydrocodone was achieved in *S. cerevisiae* (13) by combining enzymes from plants, mammals, bacteria, and the yeast itself. Moreover, high-level synthesis of tetrahydroisoquinoline alkaloids has been achieved using P450s and eukaryotic enzyme expression (14).

However, applications of *S. cerevisiae* face challenges. *S. cerevisiae* has a slower growth rate than prokaryotes such as *Escherichia coli* and *Vibrio natriegens* (15, 16). The growth rate of the *S. cerevisiae* model strain S288C is 0.42 h^−1^ or lower than those of common *E. coli* strains such as K12 and BL21 (0.63–0.68 h^−1^) (17, 18). In biosynthesis, the slow growth rate of *S. cerevisiae* is a limiting factor for its efficiency. *S. cerevisiae* is a good chassis for producing precursors of the anti-cancer drug paclitaxel, but its slow growth rate limits further increases in paclitaxel production. Researchers replaced slow-growing *S. cerevisiae* with fast-growing prokaryotic *E. coli* to produce a precursor taxadiene efficiently in a consortium, successfully solving the problem of low efficiency in oxidation taxane production (19). A fast growth rate improved the biomass growth, leading to an efficient performance of product formation. Moreover, strains with strong robustness exhibit better growth and production in different environments, which improves the efficiency and output value of industrial fermentation (2, 20). However, fast growth and robustness are difficult to obtain because they are complex processes, involve multigene control, and are host-endowed (21). Currently, robustness and growth rate can only be improved by irrational adaptive evolution. The tolerated lactic acid concentration in *S. cerevisiae* only increased from 1.3% (wt/vol) to 3.8% (wt/vol) after adaptive evolution (22). Therefore, it is urgently necessary to reveal a fast-growing robust *S. cerevisiae* as a chassis directly.

The genetic manipulation toolbox determines the modification efficiency of metabolic engineering. Genetic manipulation is challenging because of the presence of alleles in diploid industrial strains. Manipulation efficiency can be improved by isolating a stable haploid (23). Eco-friendly auxotrophs are commonly used in *S. cerevisiae* instead of antibiotic markers to avoid strain heterogeneity during industrial production (24, 25). Diverse genomic manipulation tools have already been used in *S. cerevisiae*. The Cre/*loxP* system is a traditional and highly compatible system with diverse tools (26). It has been used successfully in engineering and synthesizing the smallest yeast genome (27) and systematically increased β-carotene production (28). The CRISPR/Cas9 genome-editing system (29) has developed rapidly owing to its modernity and efficiency. A more effective GTR-CRISPR system enables the simultaneous disruption of 8 genes with 87% efficiency and a 30-fold increase in free fatty acid production in 10 days (30).

Genome-level knowledge of metabolic networks including genomic information and physiological characteristics is necessary in the field of synthetic biology. Multi-omics analyses have been used to define the genetic manipulation strategies for metabolic engineering. For example, a model of *Rhodotorula toruloides* was used to predict potential targets for the improved production of carotenoids and linolenic acid (31). Meanwhile, multi-omics analyses can help uncover new metabolic mechanisms and critical nodes, such as how *S. cerevisiae* responds to hypoxic stress by increasing glycerol phospholipid metabolism (32). Moreover, in-depth analysis of multi-omics data could provide components for synthetic biology, such as promoter libraries (33) and catalytic elements (34). In addition to revealing strain characteristics, multi-omics analysis can reveal genome-wide information. Genome-scale metabolic models have been used to comprehensively describe cell metabolism (31, 35, 36). For example, a model of *Bacillus*, *i*JA1121, was used to reveal the effects of different carbon sources on fatty acid synthesis and accumulation (35).

In the present study, we comprehensively characterized *S. cerevisiae* strain XP, a fast-growing strain with high glucose concentration tolerance that can grow under low pH (pH ≤ 3), 30% glucose concentration, and oligotrophic conditions. A genetic manipulation toolbox was developed using *URA3* auxotroph and haploid strains, Cre/*loxP*-based knocking-out method, and CRISPR/Cas9 genome editing tools to enhance its feasibility. Whole genomic, metabolomic, and transcriptomic data for this strain were analyzed in an integrated manner to reveal genome-level information of the metabolic network and explore the mechanism of rapid growth and robustness. To further verify the application potential of the chassis, we successfully constructed a genetically engineered strain for high l-lactic acid production based on its characteristics and optimized fermentation conditions.

## RESULTS

### Robustness and growth rate reveal the superiority of strain XP as a chassis

**Fig 1:**
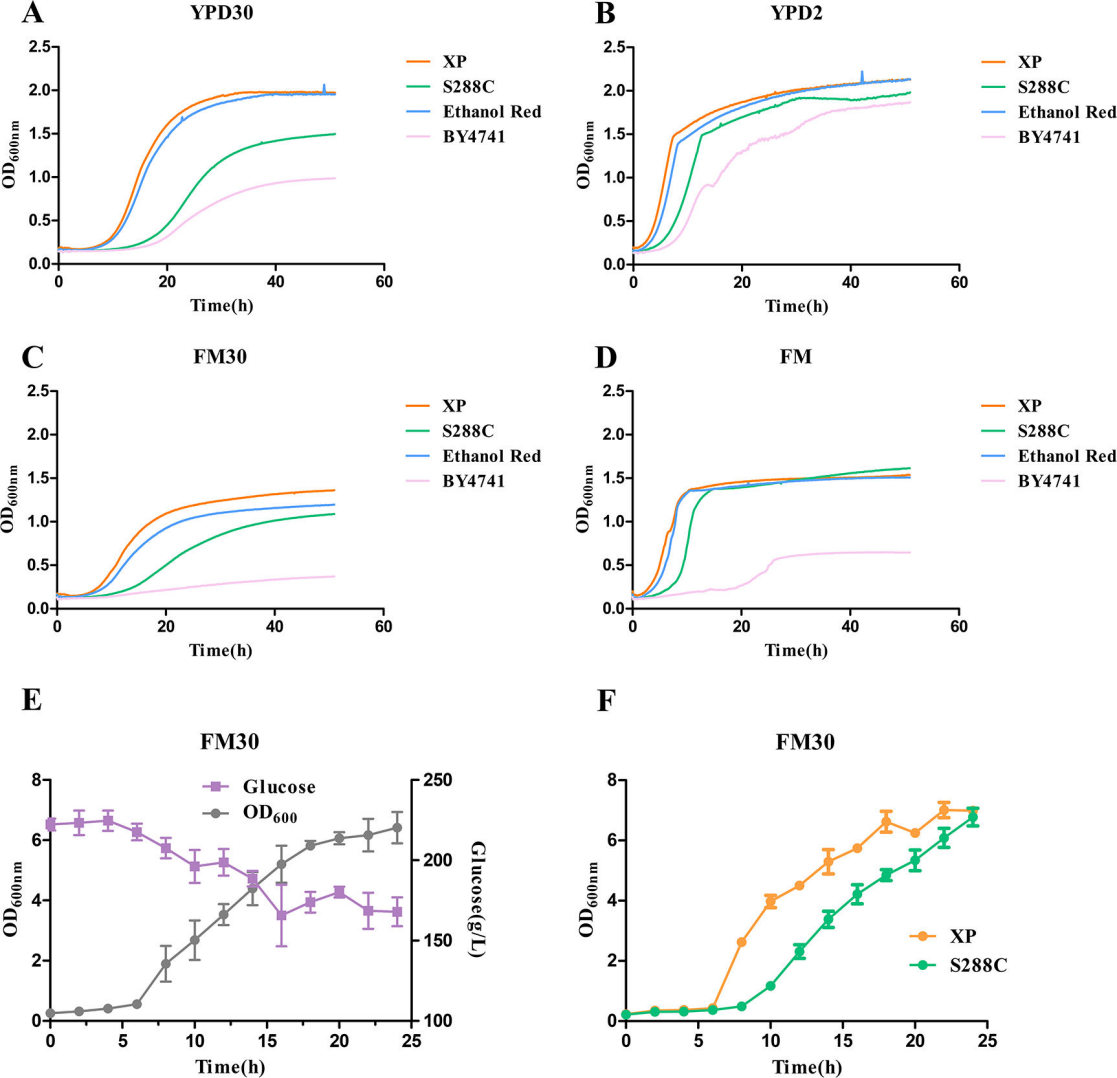
Growth rate of *S. cerevisiae* in four different mediums and fermentation culture. (**A**) Culture of *S. cerevisiae* in YPD30 medium by Bioscreen C. (**B**) Culture of *S. cerevisiae* in YPD2 medium by Bioscreen C. (**C**) Culture of *S. cerevisiae* in FM30 medium by Bioscreen C. (**D**) Culture of *S. cerevisiae* in FM medium by Bioscreen C. (**E**) *S. cerevisiae* strain XP growth and sugar tolerance in FM30 medium in 250 mL flasks. (**F**) Growth of *S. cerevisiae* strain XP and *S. cerevisiae* strain S288C in FM30 medium by fermentation culture in 250 mL flasks. Orange: *S. cerevisiae* strain XP; green: *S. cerevisiae* strain S288C; blue: *S. cerevisiae* strain Ethanol Red; pink: *S. cerevisiae* strain BY4741. Circle: OD_600nm_; square: glucose concentration. Data are the means from three parallel experiments. Error bars indicate standard deviations from three parallel experiments.

A fast-growing *S. cerevisiae* strain XP, which was screened from the soil, was used for the study owing to its possible superiority in industrial applications. Compared with the research model strains S288C and BY4741 and the industrial strain Ethanol Red, strain XP grew faster in nutrient-rich and oligotrophic media, or at high and low sugar concentrations in our automated microbiology growth curve analysis (Fig. 1A to D). In the early stages of growth, strain XP had a shorter lag period than other strains. It grew faster in the logarithmic phase and had higher biomass at 24 h. Compared with strains S288C, BY4741, and Ethanol Red, our chassis strain XP appears to be superior owing to its rapid growth rate and robustness.

The doubling time during the logarithmic growth phase was used to quantify the growth differences under different culture conditions (Table 1). The doubling time of strain XP was the shortest among that of the strains grown in the YP30, YPD2, FM30, and FM media, at 79.12 (±1.211), 43.61 (±0.8541), 84.04 (±0.8165), and 45.66 (±1.971) min, respectively. The doubling time of strain XP was only half of strain S288C in the YPD2 medium and 10.06 min faster than the industrial strain Ethanol Red. A comparison of the doubling times of strain XP in FM and YPD2 media showed that strain XP had good oligotrophic tolerance. Compared to the doubling time in YPD30 and YPD2 media, that of strain XP in FM30 and FM media was slightly inhibited under high sugar concentrations, but its doubling time was still significantly shorter than other strains in our study (Table 1; Table S4).

**TABLE 1:** Fitting of *S. cerevisiae* doubling time in four different media

Medium	Doubling time (min)			
	Strain XP	Strain S288C	Strain Ethanol Red^*a*^	Strain BY4741
YPD2	43.61 (±0.8541)	84.95 (±0.5570)	53.67 (±0.2335)	70.87 (±4.038)
YPD30	79.12 (±1.211)	153.8 (±1.705)	89.69 (±0.7954)	186.8 (±0.7755)
FM	45.66 (±1.971)	77.69 (±7.346)	68.42 (±2.721)	155.7 (±28.83)
FM30	84.04 (±0.8165)	193.4 (±3.969)	110.7 (±0.7520)	345.9 (±6.975)

^*a*^ Ethanol Red is a widely employed yeast in ethanol production in America (37).

Strains XP and S288C were scale up in the FM30 medium by shaking flask fermentation to simulate industrial conditions and test their growth superiority (Fig. 1E and F). Strain XP consumed glucose stably, and the OD_600_ value reached 6.99 at 24 h. Although their OD_600_ values were similar at 24 h, the OD_600_ values of strain XP during the fermentation process were higher than those of strain S288C. These results suggest the stable and fast growth superiority of strain XP during the culture process.

### Whole-genome alignment identifies chromosomal and mitochondrial differences

**Fig 2:**
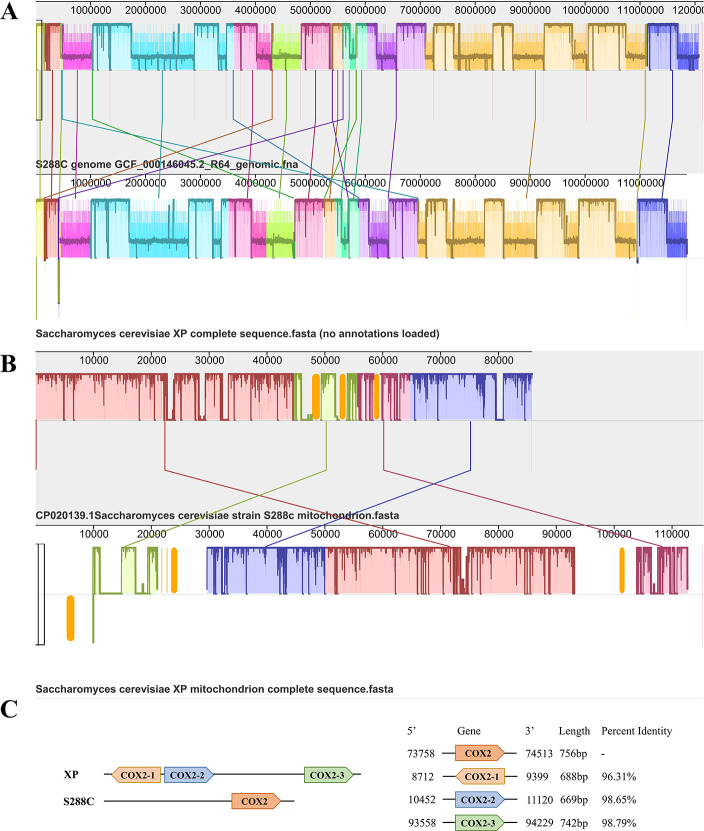
Genome alignment analysis. Each sequence is represented by one horizontal panel of blocks. Each colored block represents a region of sequence that aligns to part of another genome and is presumably homologous and free from internal rearrangements. LCBs (locally collinear blocks) >5,000 bp. (**A**) Chromosome genome alignment analysis. (**B**) Mitochondrial genome alignment analysis. (**C**) Distribution and homology of *COX2* on chromosome in *S. cerevisiae* strain XP and *S. cerevisiae* strain S288C.

To understand the genetic background, we obtained the whole-genome sequence of strain XP (Fig. 2) and uploaded the genome sequence of our strain XP to NCBI (https://www.ncbi.nlm.nih.gov/) with accession numbers CP080603–CP080619, which includes the mitochondrial genome sequence. We used AUGUSTUS to predict 6,368 ORFs in strain XP. Compared to strains XP and S288C, there were 67,264 single-nucleotide polymorphisms (Table 2). Genome alignment analysis of strains XP and S288C showed that the locally collinear blocks (>5,000 bp) between the two strains were not distinctly different at the chromosomal level (Fig. 2A). However, comparison of the mitochondrial genomes revealed many structural variations, including translocations and deletions (Fig. 2B). The mitochondrial DNA (mtDNA) length of strain XP was 115,450 bp, which is 34.6% longer than that of S288C. We wanted to identify specific genes in the mitochondria of strain XP, but all predicted ORFs can be found in the mitochondrion of S288C. The mitochondrial genome of strain XP was also compared with existing mitochondrial genomes in the NCBI database, with the closest relative mitochondrial genome *S. cerevisiae* YJM1389 having 99.3% identity. Interestingly, three *COX2* genes, encoding the Cu^2+^ sites in cytochrome *c* complex IV, were annotated on the mitochondria, with 96.3%, 98.7%, and 98.8% identities, respectively, compared with the single *COX2* in S288C (Fig. 2C).

**TABLE 2:** The substitutions of SNPs between the chromosome of strains XP and S288C

		Strain XP				Total SNPs
	Base	A	C	T	G	
Strain S288C	A	–	2,331	2,794	12,256	67,264
	C	2,175	–	12,273	1,805	
	T	2,901	12,277	–	2,297	
	G	12,117	1,973	2,065	–	

### Metabolomic analysis elucidates core pathways to fast growth and high glucose concentration

The metabolites in the FM and FM30 media were extracted to simulate oligotrophic and high glucose concentration fermentation conditions for exploring the differences between the different strains and glucose concentrations. The samples were then divided into four groups. The two groups were compared within the same strains at different glucose concentrations (S288C: S2 VS S30, XP: X2 VS X30), and two groups were compared between different strains at the same glucose concentration (low glucose condition: S2 VS X2, high glucose condition: S30 VS X30). Differences among the samples were visualized using OPLS-DA (Fig. S2). We counted the same differential transcripts and differential metabolites in each group of comparisons, and heatmaps showed that the main differential metabolites were consistent among samples (Fig. S3 and S4). Both the X2 VS S2 group and the X2 VS X30 group contained a set of data that was inconsistent with other biological replicates due to errors in sampling points. Therefore, we excluded the inconsistent data and selected the remaining points for analysis (Fig. S4). We compared the effects of improving glucose concentrations and identified 70 differential metabolites in strain S288C and 95 differential metabolites in strain XP. We found 48 common differential metabolites, mainly concentrated in starch and sucrose metabolism, nicotinate and nicotinamide metabolism, and amino sugar and nucleotide sugar metabolism (Fig. S6), among others. Additionally, we compared the differences between XP and S288C under high glucose concentrations and found a few differential metabolites. We found 68 significantly differential metabolites between strains XP and S288C when they were cultured in the FM30 medium. The 25 most differential functions were enriched through the functional enrichment of metabolites, including glycine, serine, and threonine metabolism, d-glutamine and d-glutamate metabolism, and amino sugar, and nucleotide sugar metabolism (Fig. S7).

To identify more differential metabolites, a metID algorithm with a few standards in-house database used the MS/MS spectrum for KEGG-based amplification identification. The number of differential metabolites amplified by the metID algorithm was significantly improved to 239 in positive ion mode and 375 in negative ion mode. Overall, the results provide valuable insights into the metabolic differences between these two strains and the effects of high glucose concentration on their metabolic profiles. These results were used for subsequent multi-omics analyses.

### Transcriptomic analysis indicates roles of energy metabolism on cell growth and multigene control robustness

Transcripts were also compared between strains XP and S288C in FM and FM30 media, respectively, to confirm transcriptional differences. First, we performed quality control of sequencing saturation, sequencing coverage, coverage distribution of different reads in the transcriptomic data, and sample consistency. The main differentially expressed genes (DEGs) were consistent with the heatmaps (Fig. S5). When the glucose concentration increased from 2% to 30%, strain XP had fewer DEGs than S288C. This suggests that strain XP has adapted to a high glucose environment and, therefore, responds less to stress. We mainly focused on the difference between strains XP and S288C in FM30 medium. We found 953 DEGs, of which 508 were upregulated and 445 were downregulated (Fig. S8C). The significantly different metabolism pathways were sulfur metabolism, meiosis, thiamine metabolism, snare interactions in vesicular transport, lysine biosynthesis, MAPK signaling pathway, and ABC transporters (Fig. S8A and S8B). Sulfur metabolism and ABC transporters associated with energy metabolism were upregulated, and the MAPK signaling pathway related to pressure sensing was downregulated. *MET5, MET10, MET16*, and *MET22* in sulfur metabolism were upregulated by 12.68-, 11.09-, 13.22-, and 7.38-fold, respectively (Fig. S8D). Moreover, *SKP2*, which inhibits *MET14* (38, 39), was not detected in strain XP. These DEGs indicated that transcription of the entire sulfur metabolism pathway was upregulated. Thiamine metabolism is downstream of sulfur metabolism and provides cofactors for cell growth and metabolism (40). *THI5*, *THI20*, *THI21*, *THI6,* and *THI80* in the thiamine synthesis pathway were upregulated by 154.47-, 9.89-, 7.08-, 3.60-, and 2.25-fold, respectively (Fig. S8D). Thus, we inferred that thiamine metabolism transcription was also upregulated. We also found that the individual differentially upregulated DEGs were mainly involved in cell wall synthesis and energy metabolism. *AWA1* and *ACON* were upregulated by 31.38- and 32.55-fold, respectively (Fig. S8D). The most significantly downregulated DEGs were enriched for oxidative stress and cell division, including *SCC3*, *YLL066C*, and *SDO1L*, which were downregulated by 57.29-, 16.32-, and 11.29-fold, respectively (Fig. S8D). Additionally, *FHP, CATT*, and *TSL1* associated with robustness were downregulated by 20.16-, 13.82-, and 18.88-fold, respectively (Fig. S8D). The specific functions of the DEGs are listed in Table S5.

### Multi-omics analyses construct comprehensive global knowledge of chassis XP

**Fig 3:**
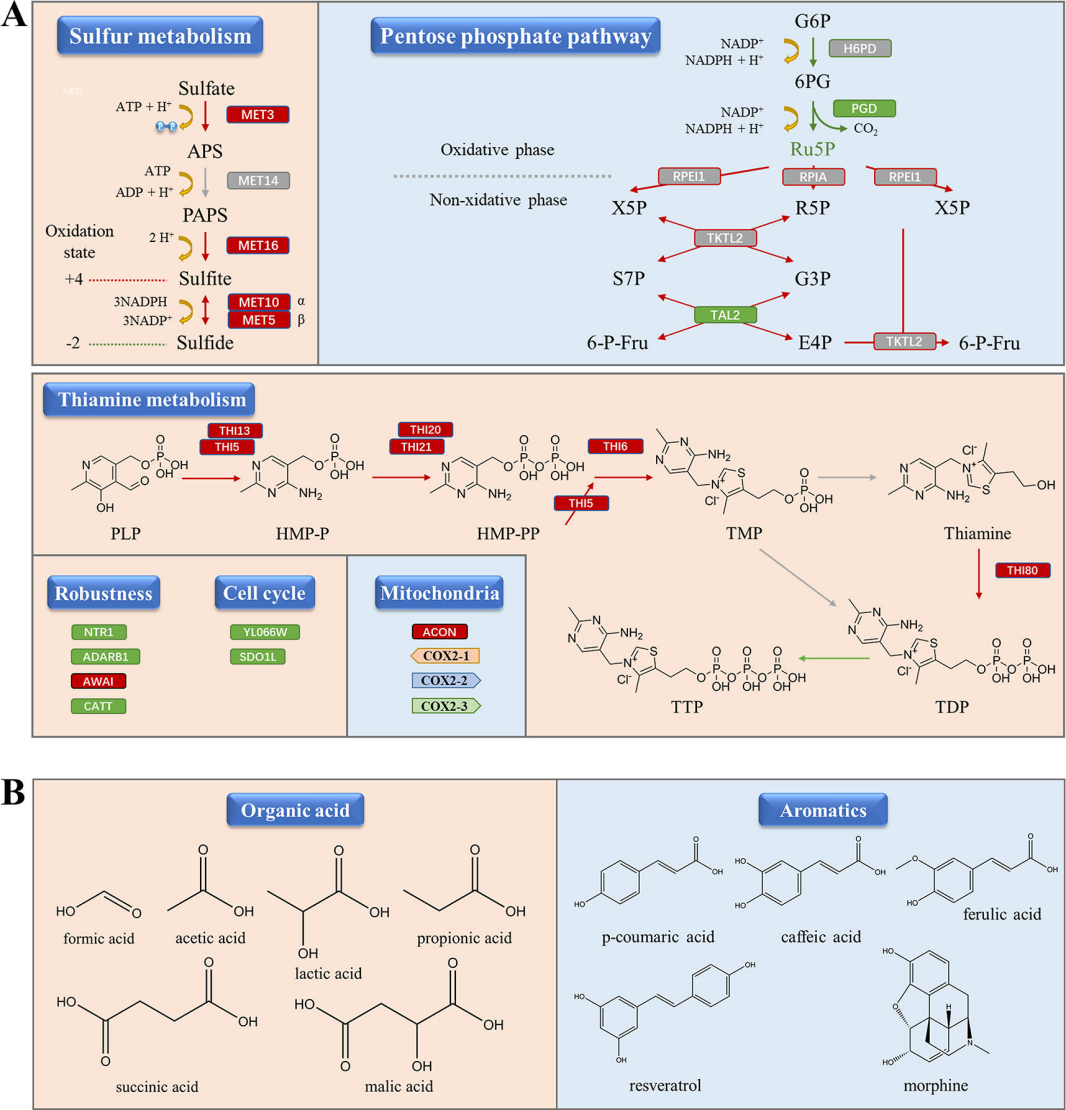
Global knowledge of metabolic networks in *S. cerevisiae* strain XP. (**A**) Changes of the pathway and characteristics in X30 VS S30. In sulfur metabolism, APS: adenylyl sulfate; PAPS: 3′-phosphoadenylyl sulfate. In pentose phosphate pathway, G6P: β-d-glucose-6P; 6PG: β-d-gluconate-6P; Ru5P: β-d-ribulose-5P; R5P: β-d-ribose-5P; X5P: β-d-xylulose-5P; S7P: d-sedoheptulose 7-phosphate; G3P: d-glyceraldehyde 3-phosphate; 6-P-Fru: β-d-fructose-6P; E4P: d-erythrose-4P. In thiamine metabolism, PLP: pyridoxal phosphate; HMP-P: 4-amino-5-hydroxymethyl-2-methylpyrimidine phosphate; HMP-PP: 4-amino-5-hydroxymethyl-2-methylpyrimidine diphosphate; TMP: thiamin monophosphate; TDP: thiamin diphosphate; TTP: thiamin triphosphate. Red arrow: increased pathways inferred by multi-omics analysis. Green arrow: decreased pathways inferred by multi-omics analysis. Gray arrow: pathways without changes inferred by multi-omics analysis. Red box: upregulated genes inferred by transcriptome analysis. Green box: downregulated genes inferred by transcriptome analysis. Gray box: genes without changes inferred by transcriptome analysis. Red background corresponds to organic acid production in Fig. 3B. Blue background corresponds to aromatics in Fig. 3B. (**B**) Appropriate products for *S. cerevisiae* strain XP.

Transcription and metabolism are inseparable processes of organisms. To further investigate the metabolic differences between the two strains, we conducted multi-omics analyses of the transcriptomic and metabolomic data between strains XP and S288C in FM30 medium (Fig. S9). Gene ontology (GO) enrichment of differential metabolites and DEGs matched the crucial differential metabolic pathways (Fig. S10 to S12). A large number of differential pathways were upregulated under high glucose concentrations in strain XP (Fig. S9A). Mainly, pathways were matched with primary metabolism, while we focused on the pentose phosphate pathway (PPP) and carbon metabolism. Interestingly, the oxidative phase was downregulated in the PPP, whereas the non-oxidative phase was upregulated (Fig. 3A). Data related to growth were analyzed in detail (Fig. S9B). The majority of upregulated metabolites and transcripts were related to carbon metabolism and amino acids, with spermidine, 4-guanidinobutanoate, and 2-oxoglutarate showing the most significant increased metabolites. *TAL2*, *ALDH3A2*, *CTR9*, *LY6E*, and *ADARB1* were downregulated the most. They are primarily transcription factors and regulatory proteins. Moreover, *NTR1* was upregulated, promoting a nitrogen-containing nutrient transport system. The function of each gene is listed in Table S6. The analyses of the pentose phosphate pathway, latent metabolites, and DEGs were corroborated with the previous analyses, establishing comprehensive global knowledge of the metabolic network.

### Genetic manipulation toolbox gives chassis XP operability

Industrial strains require efficient production performance, but their genetic manipulation limits their application in genetic engineering. To conveniently manipulate the *S. cerevisiae* strain XP, we constructed a genetic manipulation toolbox using *URA3* auxotroph and haploid strains, Cre/*loxP*-based knock-out method, and CRISPR/Cas9 genome editing tools to make the manipulation more convenient and effective.

**Fig 4:**
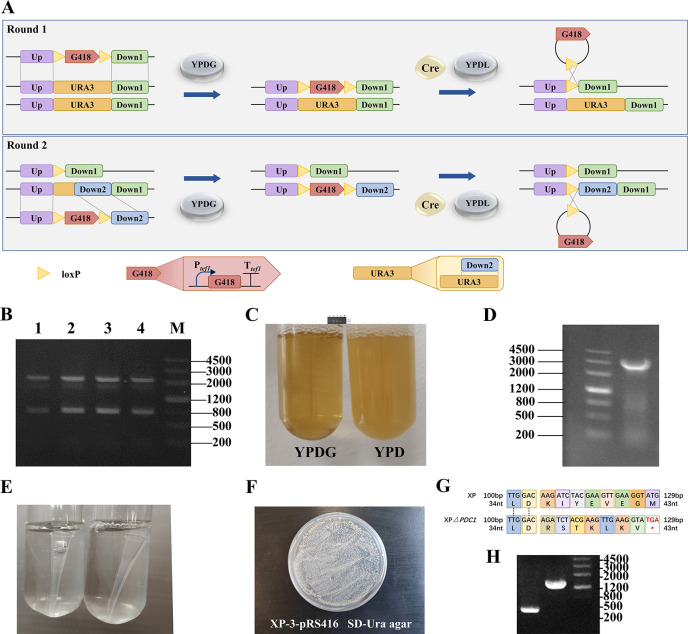
Genetic manipulation box construction. (**A**) Gene knockout flow chart by Cre/*loxP* system. (**B**) Gene knockout by Cre/*loxP* system: round 1 PCR verification. 1–4: transformant knockout homology arm verification. Homologous fragment and *URA3*: about 2 kb; *URA3*: 804 bp; M: marker III. (**C**) G418 resistance verification. Left: YPDG; right: YPD. (**D**) Gene knockout by Cre/*loxP* system: round 2 PCR verification. 1, marker III; 2, KO-URA3-2. (**E**) Uracil deficiency verification. Cultured in SD-Ura. (**F**) Plasmid transformation verification. pRS416 carries the URA3 gene to cover uracil deficiency. (**G**) Amino acid changes after editing with CRISPR/Cas9. * represents the stop codon. (**H**) Gene knockout with homologous recombination by CRISPR/Cas9 system. 1–2: transformant knockout homology arm verification, homologous fragment: 400 bp; *GPD2*:1323 bp; M: marker III.

Strain XP is a wild-type strain without auxotrophs that could only be screened with antibiotics. Due to its inability to grow with inactivated 5-phosphate decarboxylase (24, 25), we used the Cre/*loxP* system to knock out *URA3*-encoding orotidine 5-phosphate decarboxylase to construct the uracil auxotroph strain XP-3 (Fig. 4A). The first knockout box KO-*URA3*-1 was successfully transformed and bound to the genomic DNA resulting in G418 resistance (Fig. 4B and C). Subsequently, we used the second knockout box KO-*URA3*-2, to knockout another *URA3* allele (Fig. 4D). After two knockout rounds, the strains could not grow in SD-Ura medium (Fig. 4E), indicating that *URA3* alleles were inactivated. The Cre/*loxP* system was successfully applied to insert and delete the genes and the knockout strain was named XP-3. We transformed pRS416 and successfully obtained correct transformants on SD-Ura agar (Fig. 4F), verifying the screening function of strain XP-3.

Owing to the modernity and efficiency of CRISPR/Cas9, we expect it to be implemented in strain XP, which would enable a variety of options for the use of the genetic manipulation toolbox. We transformed pML104-*PDC1* into the strain and sequenced the non-homologous end joining (NHEJ) mismatch. Because of mismatches in single bases, the termination codon appeared early, causing transcription to cease prematurely and inactivating *PDC1*, which codes for pyruvate decarboxylase (Fig. 4G). In parallel, *GPD2* was successfully knocked out by fragment deletion with the homologous recombination (HR) using a double plasmid system (Fig. 4H).

**Fig 5:**
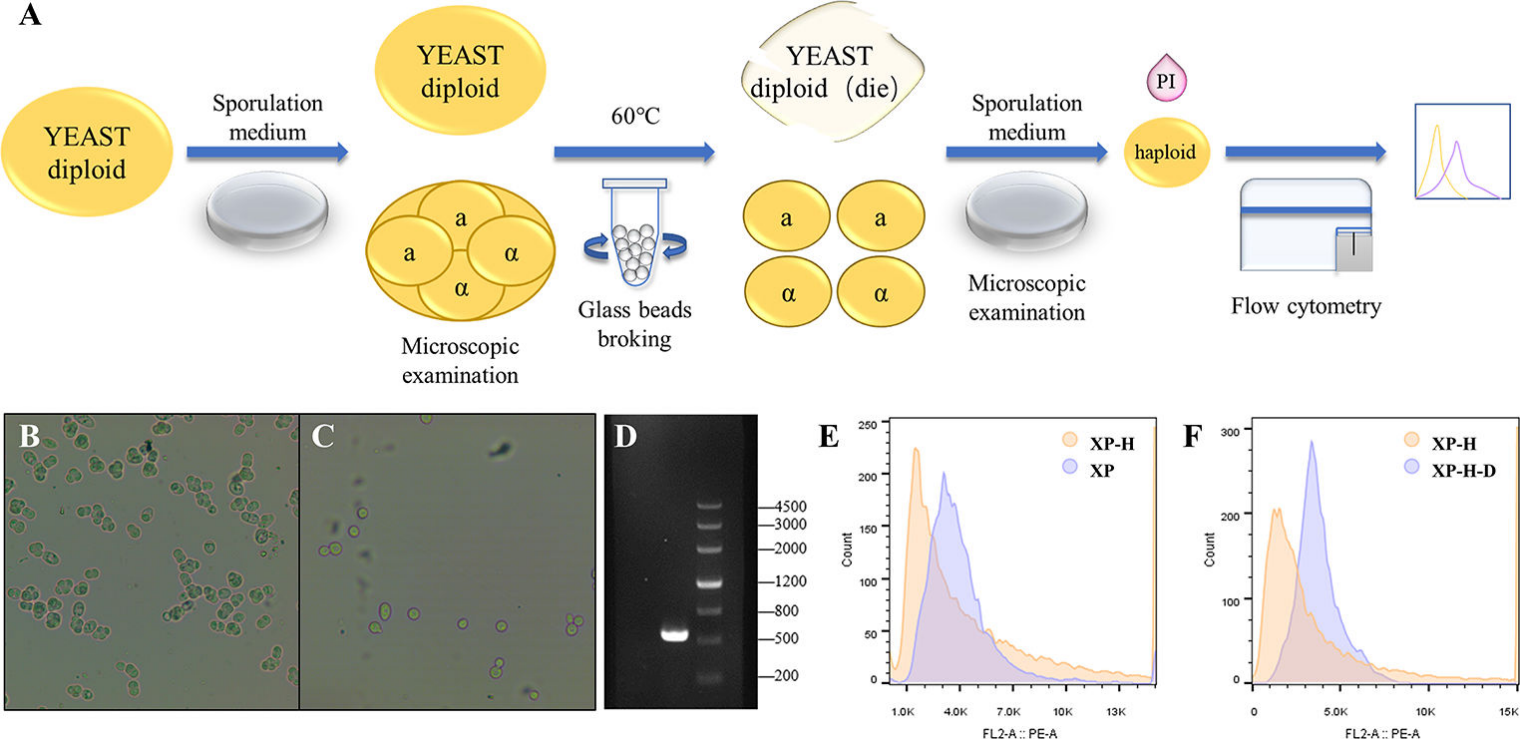
Haploid construction and ploidy transformation. (**A**) Haploid construction flow chart. (**B**) Sporulation of *S. cerevisiae* strain XP, 25°C 8 days. (**C**) Sporulation of haploid *S. cerevisiae* strain XP-H, 25°C 8 days. (**D**) Mating type PCR verification. 1: strain XP-H mating type gene *MATa* pcr fragments: 544 bp; 2: marker III. (**E**) The DNA content analysis of XP-H by flow cytometry (PI fluorescent staining). Orange: *S. cerevisiae* haploid strain XP-H. Purple: *S. cerevisiae* diploid strain XP. (**F**) The DNA content analysis of XP-H-D by flow cytometry (PI fluorescent staining). Orange: *S. cerevisiae* haploid strain XP-H. Purple: *S. cerevisiae* diploid strain XP-H-D.

Haploids were constructed to simplify the genetic manipulation of diploid yeast strains (Fig. 5A). We cultured strains XP and XP-3 at 25°C and observed tetrads to ensure sporulation (Fig. 5B). After inactivation of the parental cells, the spores of strains XP and XP-3 were sporulated and cultured again. Although no tetrads were found, spheres were observed, proving that haploids were constructed successfully (Fig. 5C). To confirm the construction of the haploid, we performed a PCR verification of *MATa/α* and only observed a *MATa* electrophoretic band (Fig. 5D). The haploids were named XP-H and XP-H3, respectively. The DNA content of XP-H was only half of that of XP (Fig. 5E), which further verified the successful construction of the haploid. We also constructed a mating method to ensure that haploids could revert to diploids. After expressing the functional HO gene in XP-H, the diploid strain XP-H-D was verified to have twice the DNA content of XP-H, which was nearly equal to that of XP (Fig. 5F).

### l-Lactic acid production by *S. cerevisiae* XP

**Fig 6:**
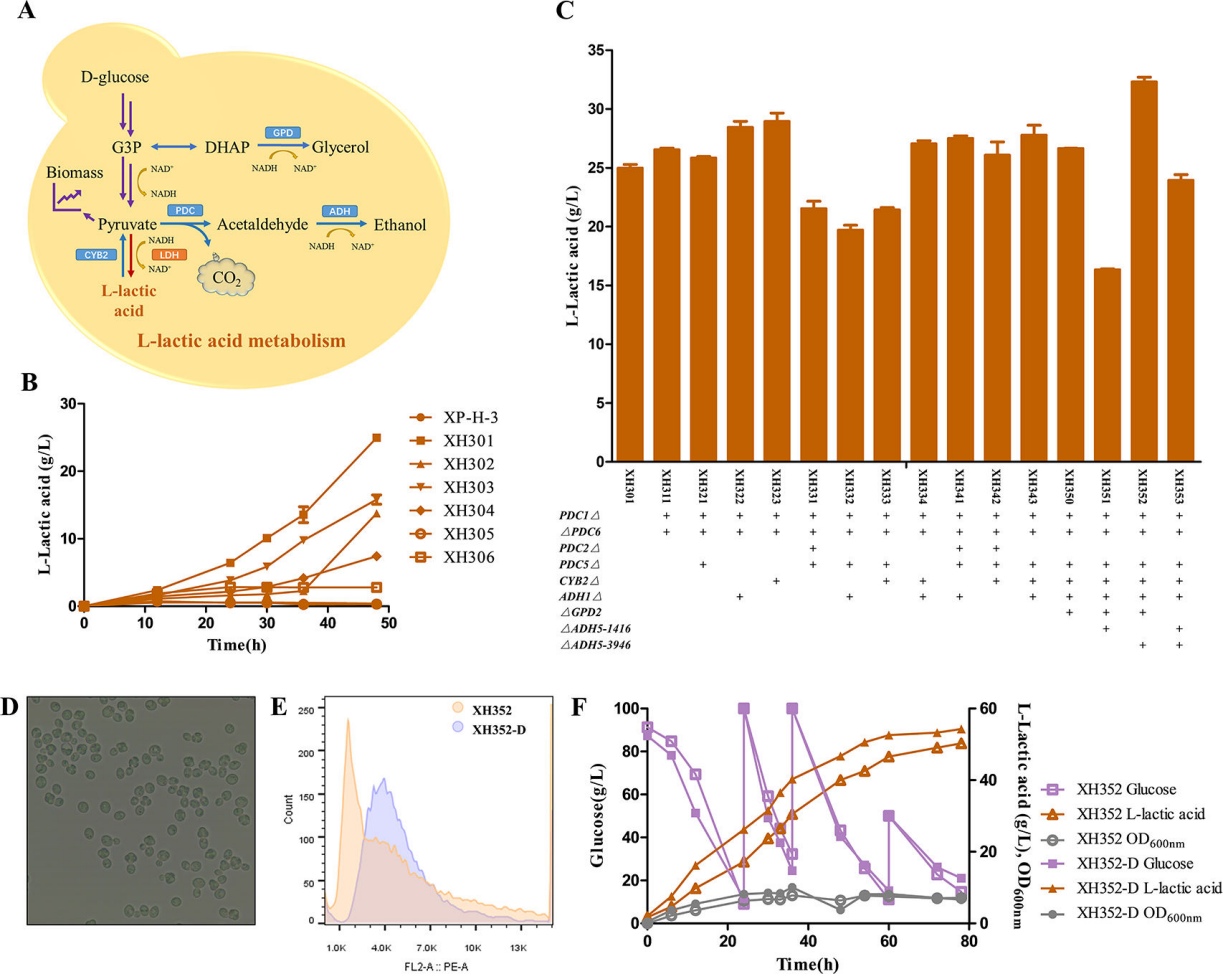
l-Lactic acid fermentation. (**A**) l-lactic acid metabolism pathway in *S. cerevisiae* XP. (**B**) LDH screening. Data are the means from three parallel experiments. Error bars indicate standard deviations from three parallel experiments. (**C**) l-lactic acid production in genetic engineering strains. Data are the means from three parallel experiments. Error bars indicate standard deviations from three parallel experiments. △*gene*: gene knockout by HR. *gene*△: gene blocking by NHEJ. (**D**) Microscopic examination of sporulation of XH352-D. (**E**) The DNA content analysis of XH352-D by flow cytometry (PI fluorescent staining). Orange: *S. cerevisiae* haploid strain XH352. Purple: *S. cerevisiae* diploid strain XH352-D. (**F**) Fermentation of strain XH352 and XH352-D in 5L fermenters.

The l-LDH catalyzed pyruvate to l-lactic acid (Fig. 6A). We did not detect l-lactic acid accumulation during the flask fermentation of *S. cerevisiae* XP (Fig. 6B). To screen suitable l-lactic acid expression elements, the genes *BcLDH_H-1_*, *BcLDH_2-6_*, *BtLDH*, *LhLDH*, *LmLDH,* and *RoLDH*, which encode l-LDHs from *Bacillus coagulans*, *Bos taurus*, *Lactobacillus helveticus*, *Leuconostoc mesenteroides,* and *Rhizopus oryzae*, respectively, were expressed in *S. cerevisiae* XP to construct strains XH301, XH302, XH303, XH304, XH305, and XH306. These strains were fermented in shake flasks for 48 h to measure l-lactic acid production (Fig. 6B). XH301, which expresses l-LDHs from *Bacillus coagulans* H-1, produced the highest amount of l-lactic acid with a titer of 25.0 g/L at 48 h. The other strains, XH302, XH303, XH304, XH305, and XH306, produced l-lactic acid with titers of 13.8, 15.8, 7.4, 0.3, and 2.8 g/L at 48 h, respectively. Therefore, strain XH301 was screened as the starting strain in this study.

To concentrate the carbon flux into lactic acid production, we edited the ethanol synthesis module, the lactic acid utilization module, and the glycerol synthesis module. Ethanol was the main by-product of the l-lactic acid fermentation (Fig. 6A). To direct the carbon flux from ethanol to l-lactic acid, *PDC1, PDC5*, and *PDC6* (encoding pyruvate decarboxylase), *ADH1* (encoding alcohol dehydrogenase), and *CYB2* (encoding l-LDH for l-lactic acid utilization) were edited. *PDC1* was inserted into a TGA stop codon for inactivation and *PDC6* was knocked out by a fragment deletion in XH310 to construct strain XH311, in which the l-lactic acid concentration was increased by 6.3% to 26.5 g/L (Fig. 6C). The genes *PDC5*, *ADH1,* and *CYB2* were edited by NHEJ in various genotypes based on XH311 to improve the production of l-lactic acid. The three-gene editing strains XH322 and XH323 produced l-lactic acid with titers of 28.4 and 28.9 g/L, respectively, which increased by 7.2% and 9.1% compared with XH311. The strain XH321 produced l-lactic acid with a titer of 25.8 g/L, which was reduced by 2.6% compared with XH311. However, the four-gene editing strains XH331, XH332, and XH333 produced l-lactic acid with titers of 21.5, 19.7, and 21.4 g/L, respectively, which reduced by 18.8%, 25.8%, and 19.2% compared with XH311. The strain XH334 produced l-lactic acid with titers of 27.0 g/L, which increased by 1.9%. The five-gene editing strains XH341 and XH343 produced l-lactic acid with titers of 27.5 and 27.8 g/L, respectively, an increase of 3.6% and 4.7%. Strain XH342 produced l-lactic acid with titers of 26.1 g/L, which was reduced by 1.8%. We further knocked out *GPD2* (encoding glycerol-3-phosphate dehydrogenase) and *ADH5-1416* and *ADH5-3946* (encoding alcohol dehydrogenase) by fragment deletion in strain XH343 and in pairs to construct strains XH350, XH351, XH352, and XH353, respectively. Among these strains, XH352 showed the best l-lactic acid production with a titer of 32.3 g/L, which increased by 16.3% compared with XH343. Other strains XH350, XH351, and XH353 produced 26.6, 16.3, and 23.9 g/L l-lactic acid at 48 h.

To facilitate l-lactic acid production, we engineered the ploidy of the strain XH352. The diploid strain XH352-D was screened by microscopic examination of sporulation (Fig. 6D) and flow cytometry (Fig. 6E). Triploid strain XH352-TR and tetraploid XH352-TE were also screened in this process (Fig. S13). However, the growth of XH352-D is slower than that of XH352. In concordance, XH352-D showed an l-lactic acid titer of 28.3 g/L which was 9.4% lower than that of XH352 in the flask fermentation (Fig. S14). The reduction in biomass under shake flask conditions was likely the reason for the decreased l-lactic acid production of diploids.

### Fed-batch fermentation of l-lactic acid

To test its industrial production and stability, strain XH352 was used to scale up the production of l-lactic acid in a 5 L fermenter. The fed-batch fermentation of XH352 showed that the l-lactic acid titer, yield, and productivity reached 46.6 g/L, 0.20 g/g glucose, and 0.78 g L^−1^ h^−1^ at 60 h, respectively. Meanwhile, the strain XH352-D was also fermented in a 5 L fermenter to test the advantages of diploids. Its titer, yield, and productivity reached 52.6 g/L, 0.22 g/g glucose, and 0.87 g L^−1^ h^−1^ at 60 h, respectively, an increase of 12.9%, 11.6%, and 12.9% compared with that of XH352 (Fig. 6F). However, increasing chromosome multiples did not promote l-lactic acid production (Fig. S15). The fed-batch fermentation of XH352-TR showed that the l-lactic acid titer, yield, and productivity reached 43.3 g/L, 0.22 g/g glucose, and 0.72 g L^−1^ h^−1^ at 60 h. The titer and productivity of XH352-TR decreased by 17.7%, 1.5%, and 17.7% compared with that of XH352-D, respectively. The titer, yield, and productivity of tetraploid XH352-TE reached 44.8 g/L, 0.22 g/g glucose, and 0.75 g L^−1^ h^−1^ at 60 h, a decrease of 14.8%, 3.3%, and 14.8% compared with that of XH352-D.

### Fed-batch fermentation of l-lactic acid under low pH conditions

To test its robustness in industry, the strain XH352-D was used to scale up the production of l-lactic acid in a 5 L fermenter under different low pH values. The l-lactic acid production was inhibited with decreasing pH (Fig. S16). When the pH value was at 4, the titer, yield, and productivity reached 32.8 g/L, 0.15 g/g glucose, and 0.55 g L^−1^ h^−1^ at 60 h, respectively, a decrease of 37.6%, 34.1%, and 37.6% compared with that at pH 5. When the pH value was 3, the titer, yield, and productivity reached 19.6 g/L, 0.14 g/g glucose, and 0.32 g L^−1^ h^−1^ at 60 h, respectively, a decrease of 62.9%, 39.8%, and 62.7% compared with that at pH 5.

## DISCUSSION

The eukaryotic chassis plays a vital role in industrial production. *S. cerevisiae* is the most commonly used chassis owing to its clear genetic background and convenient genetic manipulation. Currently, *S. cerevisiae* is primarily used for food brewing and biofuel production (41). *S. cerevisiae* has also been used for the *de novo* synthesis of high-value compounds and proteins (11). Our chassis strain XP appears to be superior owing to its rapid growth rate and robustness. The doubling time was 43.61 (±0.8541), with only half of the strain S288C in the YPD medium (Table 1). Even in the fermentation medium under 30% glucose conditions, strain XP was also the fastest growing of all strains in our study. The development of strain XP into a eukaryotic chassis can potentially reduce production cycles while offering significant economic potential for industrial applications with broad prospects.

To conveniently manipulate the *S. cerevisiae* strain XP, we constructed a gene manipulation toolbox. The Cre/*loxP* system was successfully applied to insert and delete the genes (Fig. 4B to E), and the CRISPR/Cas9 system was used to inactivate the genes (Fig. 4G and H). Additionally, the uracil auxotroph strain XP-3 (Fig. 4E and F) and haploid strain XP-H (Fig. 5) were successfully constructed. These results demonstrate the effectiveness of our gene manipulation toolbox for strain XP, which includes a variety of operating methods. Multi-omics analysis has helped us to understand the genetic background and design of metabolic engineering (32, 35). We obtained the whole-genome sequence of strain XP (Fig. 2), and transcriptome and metabolome data for strains XP and S288C (Fig. S2 to S5). Analyses of these data revealed differences in mitochondrial, sulfur and thiamine metabolism, and pentose phosphate pathways between strain XP and standard strain S288C (Fig. 3A). These data are beneficial for establishing comprehensive knowledge of strain XP.

These promising results made us interested in the mechanism of fast growth and high glucose tolerance in strain XP. We noted that the non-oxidative PPP was upregulated (Fig. 3A). In this pathway, *TAL2*, a gene that catalyzes glyceraldehyde 3-phosphate (G3P) and sedoheptulose 7-phosphate (S7P) utilization, was downregulated (Fig. S8; Fig. 3A). The G3P is a node that connects the PPP and glycolysis. G3P can be used for energy metabolism (42). This suggests that carbon flux may flow into glycolysis to produce energy for cell growth. Meanwhile, we also noticed that sulfur metabolism and the ATP levels in strain XP were upregulated (Fig. 3; Fig. S8B and S18). Previous studies have reported that the strength of sulfur metabolism is positively correlated with the levels of ATP and energy utilization (43, 44). This suggests that energy utilization through sulfur metabolism may also increase the growth rate in strain XP. We observed that the thiamine metabolic pathway in strain XP was significantly stronger than that in strain S288C (Fig. 3A; Fig. S8B). Previous studies have reported that thiamine pyrophosphate (TPP) is a cofactor for key TPP-dependent enzymes in carbon metabolism (45). These enzymes maintain cell growth (46, 47). TPP also relieves the limitations of thiamine deficiency on cell growth (45). Therefore, we speculated that sufficient key metabolites also accelerate the growth of strain XP (38, 48). Combining the results of previous and present studies, it is reasonable to infer that fast-growing performance is mainly due to strong energy utilization and sufficient key metabolites. In addition, genome sequencing data showed that strain XP had longer mtDNA (Fig. 2B and C). According to a previous study, more mtDNA can accelerate the initiation of cell division (44). The multiple copies of the *COX2* gene may also contribute to the electron transport of strain XP. *ADARB1*, which may inhibit cell proliferation (49), was downregulated in strain XP (Fig. S9B). All of these results are beneficial for fast growth.

We also found that multiple factors may contribute to the host-endowed robustness of strain XP. As mentioned above, strain XP had a strong thiamine metabolic pathway (Fig. S8B and S8D). Researchers have found that TPP prevents yeast from experiencing metabolic stress (40, 50, 51). As noted, spermidine accumulated to a greater extent in strain XP (Fig. S9B). Its accumulation promotes the stability of cell membranes and walls (52). These results indicate that strain XP has stronger adaptability to pressure conditions. Moreover, we detected *FHP* and *TSL1* downregulation (Fig. S8D). These are all associated with stress perception (53, 54), suggesting that strain XP is insensitive to stress. The adaptability and insensitivity conjointly illustrate the host-endowed robustness.

It is critical to determine whether a product is appropriate for a specific chassis (16). The carbon flux to E4P was increased by the strong pentose phosphate pathway in strain XP (Fig. 3A). E4P is a precursor to aromatic compounds (55). The phenylalanine, tyrosine, and tryptophan pathways were also strengthened in strain XP (Fig. S11). These pathways are associated with the synthesis of aromatic acids such as terpenoids (11, 56) and flavonoids (57). This implies that strain XP suits the synthesis of aromatic compounds (Fig. 3B). Strain XP had stronger energy metabolism (Fig. 3A). Energy metabolic intensity is positively correlated with NADH synthesis and electron transport chain efficiency (3). This implies a strong electron transport chain and an increased NADH supply in strain XP. The synthesis of many important aromatic compounds is catalyzed by P450s, which require electron transport chain coordination and NADH supply (3). The strong electron transport chain and abundant NADH supply suggest better functional expression of P450s in strain XP. The suitable upstream pathways, abundant precursors, and strong electron transport chain suggest the advantages of aromatic compound synthesis in strain XP. We discovered that strain XP could tolerate fermentation conditions at pH 3 (Fig. S1). Simultaneously, spermidine improves the stability of cell membranes and cell walls (52), making strain XP stable under low pH conditions. Tolerance to low pH conditions also indicates that strain XP is a suitable chassis for organic acid production (Fig. 3B).

The l-LDH from *B. coagulans* was first successfully expressed and showed better synthetic ability for l-lactic acid production (Fig. 6B) than the l-LDH from other bacteria (58), fungi (59), and mammals (60) reported in *S. cerevisiae*. This provides an optional superior l-LDH that promotes l-lactic acid fermentation in other yeasts. High-cost nutrient-rich media are often used for the fermentation of l-lactic acid (61, 62). The FM10 medium is a cheap medium that contains only 0.2% yeast extract. Meanwhile, a lower pH value was maintained in our study (Fig. S16). Fermentation at low pH values decreases the use of neutralizing agents (58). This means that our strains and medium are more economical, reducing the cost of l-lactic acid fermentation. Although the highest titer in our study is 52.6 g/L (Fig. 6F), we also found that ethanol accumulated in XH352-D (Fig. S17), which consumes the carbon flux might be due to the incomplete inactivation of *PDC5* mismatch. The genes *PDC2*, *ADH2*, *ADH3*, and *ADH6* that were annotated in XP were not knocked out and only *GPD2* for glycerol synthesis and *CYB2* for l-lactic acid utilization were knocked out (Fig. 6C). These results suggested that more systematic metabolic engineering could further improve the titer, yield*,* and productivity of l-lactic acid in XH352-D.

### Conclusion

In this study, we comprehensively characterized and demonstrated the potential of a fast-growing and robust *S. cerevisiae* strain XP for chassis development. A genetic manipulation toolbox for the strain XP was successfully built. Multi-omics data were determined and analyzed in an integrated manner to characterize the strain at the multi-omics level, providing comprehensive knowledge of metabolic networks and genetic manipulation strategies for the yeast. These analyses imply that the superior performance of strain XP is mainly controlled by multiple factors, suggesting that energy metabolism and key nutrients are important for fast growth. Additionally, we successfully used the chassis XP to construct a high-producing strain, XH352-D, that can produce l-lactic acid at low pH values. Our study highlights the remarkable competence of a fast-growing and robust *S. cerevisiae* strain as a next-generation eukaryotic microbial chassis, opening the door for its feasible applications in industrial production.

## MATERIALS AND METHODS

### Strains, media, and plasmids

There are four yeast strains in this study. *S. cerevisiae* strain XP is a fast-growing laboratory strain isolated from soil samples in Shandong, which was deposited at China Center for Type Culture Collection (No. 20221048). Prof. Zhao in Shanghai Jiao Tong University provided industrial strain *S. cerevisiae* Ethanol Red (GCA_001078105.1) and standard strain *S. cerevisiae* S288C (GCF_000146045.2). The strain Ethanol Red is a widely employed yeast in the ethanol production in America (37). Prof. Hou in Shandong University provided standard haploid strain *S. cerevisiae* BY4741 (GCA_000766575.2). All strains are shown in Table S1.

There are four media in this study for growth curve determination. YPD2 medium (commonly referred to as YPD medium) and YPD30 medium were nutrient-rich media. YPD2 medium contains 10 g/L yeast extract, 20 g/L peptone, and 20 g/L glucose. YPD30 medium contains 10 g/L yeast extract, 20 g/L peptone, and 300 g/L glucose. FM medium (also referred to as FM medium) and FM30 medium were inorganic salt media, respectively. FM medium contains 1 g/L KH_2_PO_4_, 1 g/L NaCl, 0.7 g/L MgSO_4_, 4 g/L (NH_4_)_2_SO_4_, 2 g/L yeast extract, and 20 g/L glucose. FM30 medium replaces 20 g/L glucose with 300 g/L glucose. The media used for screening are as follows. YPDG contains geneticin (G418) with a final concentration of 400 µg/mL added to YPD medium for screening resistance. YPDB contains bleomycin with a final concentration of 400 µg/mL added to YPD medium. SD-Ura medium contains 1.7 g/L YNB, 5 g/L (NH_4_)_2_SO_4_, 0.77 g/L CSM-Ura, and 20 g/L glucose used to screen uracil auxotroph. Uracil with a final concentration of 20 mg/L is added to SD-Ura to cover uracil. Mcclary medium contains 1 g/L glucose, 1.8 g/L KCl, 8.2 g/L NaAc, 2.5 g/L yeast extract, and 20 g/L agar for haploid construction. YPDL medium contains 10 g/L yeast extract, 20 g/L peptone, and 20 g/L galactose. FM10 medium replaces 20 g/L glucose with 100 g/L glucose for fed-batch fermentation. There are four plasmids used in this study. pUG6 contains G418 resistance with *loxP* sites on both sides. pSH65 expresses Cre enzyme to make *loxP* site homologous recombination under the induction of galactose. pRS416 is an expression vector containing a *URA3* screening marker. pML104 expresses Cas9 and contains guide RNA expression cassette with *Bcl*I-*Swa*I cloning sites (63). All plasmids are shown in Table S2.

### Determination of doubling time fitting and high glucose tolerance

We used an Automated Microbiology Growth Curve Analysis System (Bioscreen, FP-1100-C, Finland) to determine the growth curve. Strains of XP, Ethanol Red, S288C, and BY4741 were cultivated at 30°C by inoculating 1% (vol/vol) of seed cultures in the test tube containing 5 mL YPD medium overnight, and then the cultures were activated twice with 1% (vol/vol) inoculation volume. Cultivation was conducted in a 100-well plate biological parallel with three biological duplications at 30°C. The OD_600_ value was determined every 10 min. The R package was used to fit the doubling time in each case (64). We choose the value of OD_600_ <1.0 to fit the doubling time to ensure reliability. In the actual large-scale fermentation of industrial production, the growth status was quite different from that in small systems. To determine high glucose concentration tolerance and growth rate in FM30 medium in an enlarged system, strains XP and S288C were cultivated by inoculating 1% (vol/vol) of seed cultures in 250 mL flasks, each of which contains 50 mL FM30 medium. Cultivation was conducted at a shaker speed of 200 rpm at 30°C. The OD_600_ values and glucose concentrations were determined every 2 h. The optical density was measured at 600 nm using a V‐1200 spectrophotometer (MAPADA, Shanghai, People’s Republic of China). The glucose concentrations were determined by SBA-40D biosensor analyzer (Institute of Biology, Shandong Academy of Sciences, China).

### Construction of gene editing tools

Because there were alleles in the diploid, *URA3* should be knocked out with Cre/*loxP* system (12) twice, in turn, to construct uracil auxotrophy. Primers used in this study are listed in Table S3. First, the upstream and downstream homologous fragments of *URA3* were amplified through PCR with the primers *URA3*KOup-F and *URA3*KOup-RV, *URA3*KOdown-F1 and *URA3*KOdown-RV1 from genomic DNA of strain XP, respectively. The *loxP-P*_tef_-G418-*T*_tef_-*loxP* fragment was amplified through PCR with the primers *URA3-KanMX*-F1 and *URA3-KanMX*-RV1 from pUG6. Overlap extension PCR was used to fusion these three fragments into a knockout box, named KO-*URA3*-1. Then, strain XP was transformed KO-*URA3*-1 using the LiAc transformation method and cultivated on the YPDG agar. After PCR verification of the integration of KO-*URA3*-1 on genomic DNA using primers *URA3-KanMX*-F1 and URA3-*KanMX*-RV1, strain XP was transformed pSH65 and cultivated on the YPDB agar. Cre enzyme was induced on the YPDL agar for *loxP* site recombination. *URA3*KOup-F and *URA3*KOdown-RV1 were used to verify *URA3* of one chromosome knockout. Then, pSH65 was dropped out by continuous culture to knock out *URA3* in the next turn. Second, a homologous fragment in *URA3* was amplified through PCR with the primers *URA3*KOdown-F2 and *URA3*KOdown-RV2 from the strain XP genomic DNA as another downstream template. The *loxP-P*_tef_-G418-*T*_tef_-*loxP*-2 fragment was amplified through PCR with primers *URA3-KanMX*-F2 and *URA3-KanMX*-RV2 from pUG6. Overlap extension PCR was used to fusion these two fragments and *URA3* upstream into a knockout box, named KO-*URA3*-2. Another copy of *URA3* was knocked out using the same method as the first copy. After dropping out pSH65 by continuous culture from strain XP, the knockout strain was cultivated in the SD-Ura medium to determine uracil auxotrophic, named XP-3. pRS416 was transformed into strain XP-3 by the LiAc transformation method and cultured at SD-Ura agar to determine manipulability.

CRISPR/Cas9 system containing single plasmid system (pML104) and double plasmid system (p414-TEF1p-Cas9-CYC1t and pRS42H) was established for strain XP. Guide sequences were designed by CRISPOR (http://crispor.tefor.net/crispor.py). In the single plasmid system, Primers XP*PDC1*sgRNA-F and XP*PDC1*sgRNA-RV, containing the *N*-terminal portion of the gRNA scaffold, were designed to form the sticky end of *the Bcl*I restriction site after annealing. Mixing primers were incubated at 95℃ for 2 min and naturally cooled to form a double-stranded fragment. Then, the fragment was inserted in *Bcl*I-*Swa*I sites on pML104 to produce pML104-*PDC1* for inactivating *PDC1*. Strain XP was transformed pML104-*PDC1* and single cloning was sequenced to determine inactivation due to NHEJ. In the double-plasmid system, we took the knockout of *GPD2* as an example. The pRS42H was amplified through PCR with primers XP*GPD2*sgRNA1-pRS42H-F and XP*GPD2*sgRNA1-pRS42H-R to change the 20 nt (N20) guiding sequence. The PCR products were added 1 µL *Dpn*I to digest the template fragments and then transformed to Top10 to screen the plasmid pRS42H-*GPD2*-sgRNA1 that N20 was successfully changed. The upstream and downstream donor fragments of *GPD2* were amplified by primers *GPD2*-F and *GPD2*-donor-up-RV, *GPD2*-donor-down-F, and *GPD2*-RV from the genomic DNA of strain XP, respectively. The knockout donor fragment of *GPD2* was amplified by primers *GPD2*-F and *GPD2*-RV from the upstream and downstream donor fragments. The donor fragments and the plasmids p414-TEF1p-Cas9-CYC1t and pRS42H-*GPD2*-sgRNA1 were transformed into the strain XP-H-3 together, which then was cultured on the YPD agar containing 100 µg/mL nourseothricin and 250 µg/mL hygromycin B at 30℃ for 2 days. A 400 bp fragment was amplified by the primers *GPD2*-F and *GPD2*-RV to verify the knocking out by HR. The genetic editing plasmids were dropped out by plate streaking on the YPD agar.

### Transformation of ploidy

Most spores are more robust than parental cells, and they can develop into new individuals after being separated from their parental cells (65). We used the glass bead crushing method destroying the parental cells to construct haploids. Strains XP and XP-3 were activated in the YPD medium at 30°C overnight, and the activated liquid was streaked on the Mcclary agar and cultured at 25°C for 8 days. Single colonies were picked, diluted in saline, and observed sporulation under an optical microscope. Then, the single clone under the best sporulation condition was cultivated in a 1 mL YPD medium overnight. We used two steps to kill the parental cells. First, cells were centrifuged for collection at 5,000 rpm for 5 min. One milliliter of snail enzyme solution was added and incubated in a 30°C water bath for 4 h to destroy the cell wall. Then cells were put into a 60°C water bath for 10 min to kill the parental cells. Second, we added the glass beads, sterile normal saline, and sterile liquid paraffin into the tube, shaking for 20 min to further damage the parental cells and release spores. The broken liquid was diluted 10^5^ times and cultivated on a YPD agar plate at the best sporulation temperature for 2 days. Then, the sporulation status was observed under an optical microscope. The diploid produced a tetrad, while the haploid was a sphere. The haploid mating type (*MATa*/*α*) was also verified by PCR with primers P1, P2, and P3 (*MATa*: 544 bp, *MATα*: 404 bp) (66).

Since the HO gene that encoding homothallic switching endonuclease in the laboratory strain has been mutated and lost its function, we need to restore its function by site-directed mutagenesis. First, the HO gene fragment was amplified by primers HO-F and HO-R from the genomic DNA of strain BY4741 and inserted between the *Eco*RI and *Sph*I sites on the plasmid pUC19 to construct plasmid pUC19-HO. Then, primers M1-F and M1-R were used to PCR the plasmid pUC19-HO to mutate the 667th base A to G. The amplified product was digested the PCR template with *Dpn*I and transformed into *E. coli* Top10. Primers M2-F and M2-R were used to PCR the plasmid which was sequenced correctly in the previous step to mutate the 1,424th base T to A. The amplified product was digested with *Dpn*I and transformed into *E. coli* Top10. The plasmid which contained the functional HO gene was screened by sequencing and named pUC19-HOM2. The *Xho*I-*P*_adh1_-*Bam*HI-*Eco*RI-*T*_adh1_-*Not*I fragment was synthesized by Qinglanbio company (Wuxi, China) and inserted between the *Xho*I and *Not*I sites on plasmid pRS416 to construct plasmid pRS416-*P*_adh1-_*T*_adh1_. The functional HO gene fragment was amplified by the primers HO-416-F and HO-416-RV from the plasmid pUC19-HOM2 and inserted between the *Bam*HI and *Eco*RI sites on the plasmids pRS416-*P*_adh1-_*T*_adh1_ to construct plasmid pRS416-HOM2.

The plasmid pRS416-HOM2 was transformed into *S. cerevisiae* using the LiAc transformation method and cultured on the SD-Ura agar. The single-cloning was incubated in the SD-Ura liquid medium at 30°C overnight to mate the *MATa* type haploid and the *MATα* type haploid. One band (544/404 bp) was verified in the haploid while two bands (544 and 404 bp) were verified in the diploid and polyploids by primers P1, P2, and P3. Meanwhile, the microscopic examination of tetrad was also used to determine the ploidy. To further verify the ploidy, the single cloning was cultured and activated in the YPD medium until the plate phase. Then, the cells were collected at 12,000 rpm for 1 min and dyed in a solution including 25 µg/mL propidium iodide (PI). The cells were washed in PBS buffer and determined the DNA content of the strains using flow cytometry analysis. The haploid *S. cerevisiae* BY4741 was used for the control strain.

### Genome sequencing and analysis

*S. cerevisiae* XP was inoculated from the glycerol tube into a 5 mL YPD medium with 1% (vol/vol) inoculation volume at 30°C and then activated twice for activation. The activated solution was moved to 50 mL YPD shake flasks until OD_600_ value to 0.6–0.8 to collect cells with centrifuging at 6,000 rpm for 20 min. Genomic DNA extraction was used Wizard Genomic DNA Purification Kit (Promega Company, USA). The total genomic DNA was sent to Frasergene Company (Wuhan, China) for third-generation sequencing by PacBio Platform. The complete genomic sequence was used AUGUSTUS for functional gene prediction (67). Mauve was used for alignment and SNP analysis (68).

### Metabolite extraction and analysis

The reported method was used to extract metabolites in *S. cerevisiae* (69). Strains XP and S288C were cultivated in the YPD medium at 30°C overnight and then inoculated 1% (vol/vol) to the FM and FM30 media in flasks. Cells were collected at logarithmic phase (OD_600_ = 2), named X2, X30, S2, S30, respectively. The liquid was quickly filtered with a volume of 5 mL to avoid metabolite loss and extracellular metabolite contamination. Then, the filter membrane was washed with 10 mL ddH_2_O. These processes should be completed within 30 s. If the OD_600_ values were different, sampling volume was adjusted to make the number of cells consistent. Then, the filtered cell masses attached to the filter membrane were mixed quickly with 20 mL of metabolite extraction solvent (acetonitrile/water [1:1, vol/vol] at –20°C) and were frozen with liquid nitrogen. When preparing samples, the extraction mixture was thawed on ice, vortexed for 3 min, and centrifuged at 12,000 rpm for 5 min at 4°C. The supernatants were frozen in a vacuum dry environment. Finally, the extracts were resuspended in 500 µL of fresh acetonitrile/water mixture (1:1, vol/vol) on the ice. Six biological replicates were performed for each sample.

Thermo UPLC Q-Extractive (QE) coupled with ESI ion source was applied for untargeted metabolomics data acquisition. Thermo UPLC Q Extractive (QE) Orbitrap system using ESI ion source was applied for data acquisition including full MS, PRM, and full MS–ddMS^2^ instrument methods. RP Zorbax Eclipse XBD-C18 column was applied for positive and negative mode analysis. The mobile phase was used for analysis with 0.1% formic acid in diluted water as aqueous phase A and 0.1% formic acid in pure acetonitrile as organic phase B. The LC gradient elution program was as follows: *t* = 0.0 min, 99% A; *t* = 5.0 min, 99% A; *t* = 5.5 min, 70% A; *t* = 9 min, 100% B; *t* = 11 min, 100% B; *t* = 12.1 min, 99% A.

The MS parameters of C18-ESIMS in the analysis of positive ionization mode are as follows: the mass range was set from *m*/*z* 80 to 1,000, with a spectra collection rate of 2.0 Hz and capillary voltage of 4,500 V; the gas flow rate of the nebulizer was 1.6 bar; the velocity of dry gas at 220°C was 6.0 L/min; funnel 1 and 2 radio frequencies (RFs) were set as 150 and 200 Vpp; collision-induced dissociation (CID) energy was set as 0 eV; quadrupole ion energy was 5 eV; low mass was *m*/*z* 50; collision cell energy was 7.0 eV; pre-pulse storage was 5.0 μs; collision RF ramp was from 400 to 800 Vpp, and transfer time ramp was from 50 to 100 μs.

We applied the database named metDNA for metabolomics data analysis (70). Full MS data were transformed to mzXML format by ms converter (ProteoWizard). Metabolites were amplified by metID (71).

### Transcriptome sequencing and analysis

Strains XP and S288C were cultivated with the same methods as metabolite extraction in FM and FM30 media. Cells were collected in the logarithmic phase (OD_600_ = 2) by centrifugation at 4°C, 12,000 rpm for 30 s. Then, we discarded the supernatants and quickly froze the precipitations with liquid nitrogen. Three biological replicates were performed for each sample. If the OD_600_ values were different, sampling volume was adjusted to make the number of cells consistent. The total RNA was extracted from four groups of samples for transcriptome analyses, named X2, X30, S2, and S30, respectively, based on the strains and glucose concentrations. The TRIzol (Invitrogen) method was applied to extract the total RNA in the sample; then, the DNase I (Takara) was used to remove the genomic DNA. The quality of RNA samples was detected by 2100 Bioanalyser (Agilent) and ND-2000 (NanoDrop Technologies). TruSeqTM RNA sample preparation kit (Illumina, SanDiego, CA) kit by The Majorbio Company (http://www.majorbio.com, Majorbio, Shanghai, China) was used for library construction. Illumina HiSeq X ten/NovaSeq 6000 sequencing platform was used for high-throughput sequencing with PE150 read length. SeqPrep (https://github.com/jstjohn/SeqPrep) and Sickle (https://github.com/najoshi/sickle) were applied for the trimming of raw paired-end reads and quality control. HISAT2 (http://ccb.jhu.edu/software/hisat2/index.shtml) (72) software was used for clean reads separately aligned to the reference genome with orientation mode, and StringTie (https://ccb.jhu.edu/software/stringtie/index.shtml?t=example) (73) was used to assemble mapped reads of each sample. According to differential expression analysis and functional enrichment, RSEM (http://deweylab.biostat.wisc.edu/rsem/) (74) was used to quantify gene abundances. The transcripts per million reads (TPM) method was used to identify differential expression genes (DEGs). Differential expression analysis was performed using the DESeq2/DEGseq/EdgeR (75–77) with the *Q* value ≤ 0.05. DEGs with |log2FC| > 1 and *Q* value ≤ 0.05 (DESeq2 or EdgeR)/*Q* value ≤ 0.001 (DEGseq) were considered to be significantly different expressed genes. Then functional enrichment including GO and KEGG was performed to identify which DEGs were significantly enriched in GO terms and metabolic pathways at Bonferroni-corrected *P*-value ≤ 0.05 compared with the whole-transcriptome background. Goatools (https://github.com/tanghaibao/Goatools) and KOBAS (http://kobas.cbi.pku.edu.cn/home.do) (78) were used to GO functional enrichment and KEGG pathway analysis. The program rMATS (http://rnaseq-mats.sourceforge.net/index.html) (79) was used to occur alternative splice events, including isoforms similar to the reference or comprised novel splice junctions, exon inclusion, exclusion, alternative 5′, 3′, and intron retention events.

### Co-analysis of transcription and metabolism

Transcriptomic and metabolomic data use OmicsBean (http://www.omicsbean.cn/) for data co-analysis, pathway co-enrichment, protein-protein interaction (PPI) analysis and metabolic network comparison.

### Construction of gene engineering strains for l-lactic acid production

The l-lactate dehydrogenase (l-LDH) fragments from *Bacillus coagulans* H-1, *Bacillus coagulans* 2-6, *Bos taurus, Lactobacillus helveticus, Leuconostoc mesenteroides,* and *Rhizopus oryzae* were amplified through PCR with the primers 416-*BcLDH_H-1_*-F and 416-*BcLDH_H-1_*-RV, 416-*BcLDH_2-6_*-F and 416-*BcLDH_2-6_*-RV, 416-*BtLDH*-F and 416-*BtLDH*-RV, 416-*LdLDH*-F and 416-*LdLDH*-RV, 416-*LmLDH*-F and 416-*LmLDH*-RV, 416-*RoLDH*-F and 416-*RoLDH*-RV from the plasmids that synthesized by Qinglanbio company (Wuxi, China), respectively. The ClonExpress II One Step Cloning Kit was used to insert these fragments between *Bam*HI and *Eco*RI sites on pRS416-*P*_adh1-_*T*_adh1_ to construct the l-LDH expressing plasmids. Plasmids constructed in this study are listed in Table S2.

The l-LDH-expressing vectors were transformed into *S. cerevisiae* to construct the genetic engineering strains listed in Table S1. To improve the titer of l-lactic acid, the pathways of by-products were knocked out. The genes *PDC1*, *PDC5*, *ADH1*, and *CYB2* were edited by CRISPR/Cas9 system to be inactivated with NHEJ. The gene *PDC6* was knocked out by Cre/*LoxP* system, and the genes *GPD2* and *ADH5* were knocked out by the CRISPR/Cas9 system with HR. The sequences of the primers and the sgRNAs are listed in Table S3.

### Fermentation of l-lactic acid

The genetic engineering strains were cultivated in the YPD medium at 30°C overnight and then inoculated 1% (vol/vol) to the FM10 medium with 2% (wt/vol) CaCO_3_ in static flasks. The flasks were sealed using plastic film to simulate anaerobic conditions and fermented for 48 h. For the fed-batch fermentation of l-lactic acid, the genetic engineering strains were cultivated in the YPD medium at 30°C overnight and then inoculated 1% (vol/vol) to the 500-mL flasks with 100 mL YPD media overnight to obtain seed cultures. All seed cultures were inoculated to the 5 L fermenter containing 3 L FM10 medium and cultured at 30°C, 100 rpm, and pH 5. The pH was regulated by 25% (wt/vol) Ca(OH)_2_ continuously in all fermentation processes. The glucose was fed to 100 g/L while it was almost used up.

The OD_600_ values were measured after diluting with an equal volume of 6 M HCl to neutralize CaCO_3_ and then with deionized water to the desired extent. The titers of glucose and l-lactic acid were analyzed using an SBA-40D biosensor analyzer. The titer of ethanol was analyzed by high-performance liquid chromatography 1260 (Agilent, California, USA) with a differential refractive index detector. The samples were diluted with an equal volume of 2 M H_2_SO_4_ to neutralize impurities and then with deionized water to the desired extent. Aminex HPX–87H Column (Bio-Rad, USA) was used to analyze products. The mobile phase was used for analysis with 5 mM H_2_SO_4_ in diluted water. The parameters are as follows: flow rate, 0.5 mL/min; injection volume, 10 µL; column temperature, 55°C; detection temperature, 55°C.

## Data Availability

All data needed to evaluate the conclusions in the paper are present in the paper and/or the supplemental material. The strain *S. cerevisiae* XP was deposited at China Center for Type Culture Collection (No. 20221048). The XP genome sequencing data were deposited at National Center for Biotechnology Information GenBank with the accession numbers CP080603–CP080619. The transcriptomic raw data were deposited at National Center for Biotechnology Information Sequence Read Archive with the accession number PRJNA939414. The metabolomic raw data were deposited in Zenodo open access repository.

## SUPPLEMENTAL MATERIAL

Supplemental material mbio.03196-23-s0001.docx Additional experimental details, data, and sequences.

ASM does not own the copyrights to Supplemental Material that may be linked to, or accessed through, an article. The authors have granted ASM a non-exclusive, world-wide license to publish the Supplemental Material files. Please contact the corresponding author directly for reuse.
