# Supplementary material for: A robust yeast chassis: comprehensive characterization of a fast-growing Saccharomyces cerevisiae
Source: mBio. 2024 Jan 12;15(2):e03196-23. doi: 10.1128/mbio.03196-23 (PMC10865977; doi:10.1128/mbio.03196-23)
Supplement: Supplemental material — Additional experimental details, data, and sequences. [file mbio.03196-23-s0001.docx]

**Supplementary Materials for**

**A robust yeast chassis: comprehensive characterization of a fast-growing *Saccharomyces cerevisiae***

Yangdanyu Long^1^, Xiao Han^1^, Xuanlin Meng^1^, Ping Xu^1^*, Fei Tao^1^*

^1^State Key Laboratory of Microbial Metabolism & School of Life Sciences & Biotechnology, Shanghai Jiao Tong University, Shanghai 200240, People’s Republic of China

*Corresponding author:

Mailing address for: Prof. Dr. Ping Xu: State Key Laboratory of Microbial Metabolism & School of Life Sciences and Biotechnology, Shanghai Jiao Tong University, Shanghai 200240, People’s Republic of China, E-mail: pingxu@sjtu.edu.cn, Tel: +86-21-34206647; Fax: +86-21-34206723

Mailing address for: Prof. Fei Tao, State Key Laboratory of Microbial Metabolism & School of Life Sciences and Biotechnology, Shanghai Jiao Tong University, Shanghai 200240, People’s Republic of China, E-mail: taofei@sjtu.edu.cn, Tel: +86-21-34206647, Fax: +86-21-34206647

**Supporting materials & methods**

**1. Screening process of *Saccharomyces cerevisiae* XP**

The soil sample was collected from Jinan, Shandong. The samples were processed and inoculated with FM30 medium. Cultures were cultivated at 30°C, 200 rpm for 24 hours. Then the cultures were separated by the dilution-plate method. The plates were cultivated at 30°C. Then single colonies were isolated and inoculated into FM30 medium and cultured at 30°C, 200 rpm. When the cultures were turbid, they were transferred into fresh FM30 medium again. After several generations, the large colonies on the FM30 plate were selected. Then we amplified their 18S rRNA nucleotide sequences using the primers of 18sF (AACTTAAAGGAATTGACGGAAG) and 18sR (TCCGCAGGTTCACCTACGGA) and identified them by comparing them with the 18S rRNA database. Strains identified as *Saccharomyces cerevisiae* were selected and then evaluated in FM30 media, strain XP was chosen because of its fast growth.

2. Testing the levels of ATP

Strains XP and S288C were cultivated with the same methods as metabolite extraction in the FM30 media. The ATP levels were tested by the ATP Luminescent Cell Viability Assay Kit (Yeasen Biotechnology, Shanghai) in the logarithmic phase (OD_600nm_ = 2). The luminescence detection reagents were thawing, and balanced to room temperature. Take out the cell culture plate and balance at room temperature for 10 min. Add 100 μL detection reagent to each hole of the 96-well plate then oscillate at room temperature for 2 min to promote cell lysis. Then the plate stayed at room temperature for 10 min to stabilize the luminous signal. Chemiluminescence detection was performed with a multifunctional enzyme marker. Set the corresponding parameters according to the requirements of the instrument, the detection time of each hole is 1 s. The relative vitality of cells is calculated according to the chemiluminescence reading. Testing the ATP standard curve to verify the linearity and accuracy.

**Supplementary Figures**


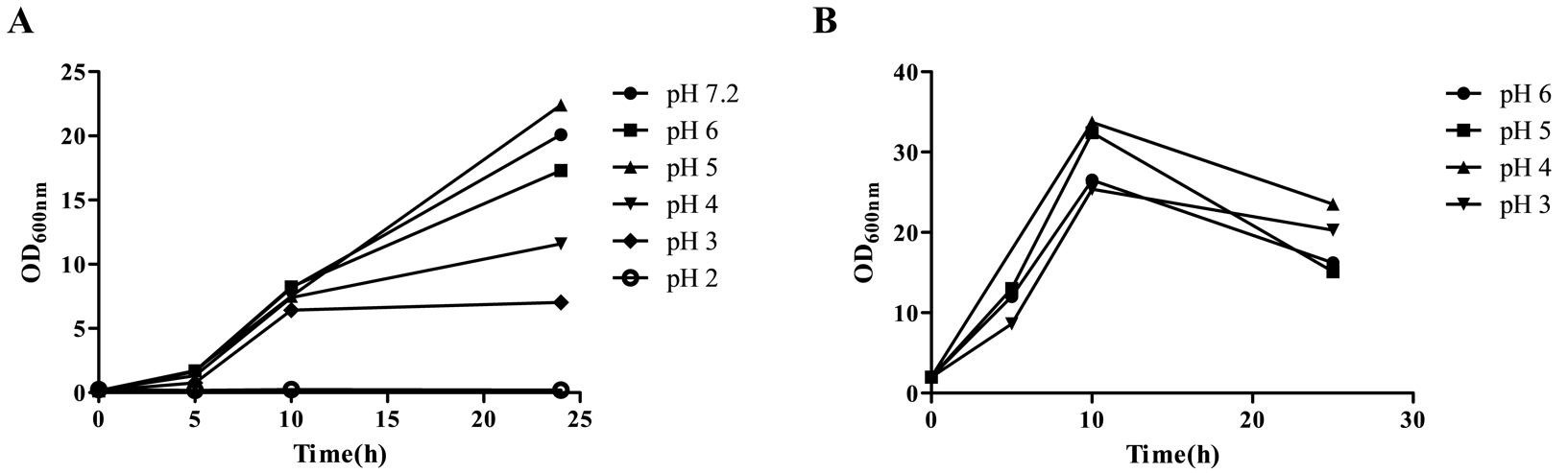


**Fig. S1. Acid tolerance of *Saccharomyces cerevisiae* XP.** (**A**) Fermentation in 250 mL YPD shake flask; adjust the initial pH with H_2_SO_4_. (**B**) Fermentation in 1 L fermenter; adjust the initial pH with H_2_SO_4_; use 10 mol/L NaOH to control pH.


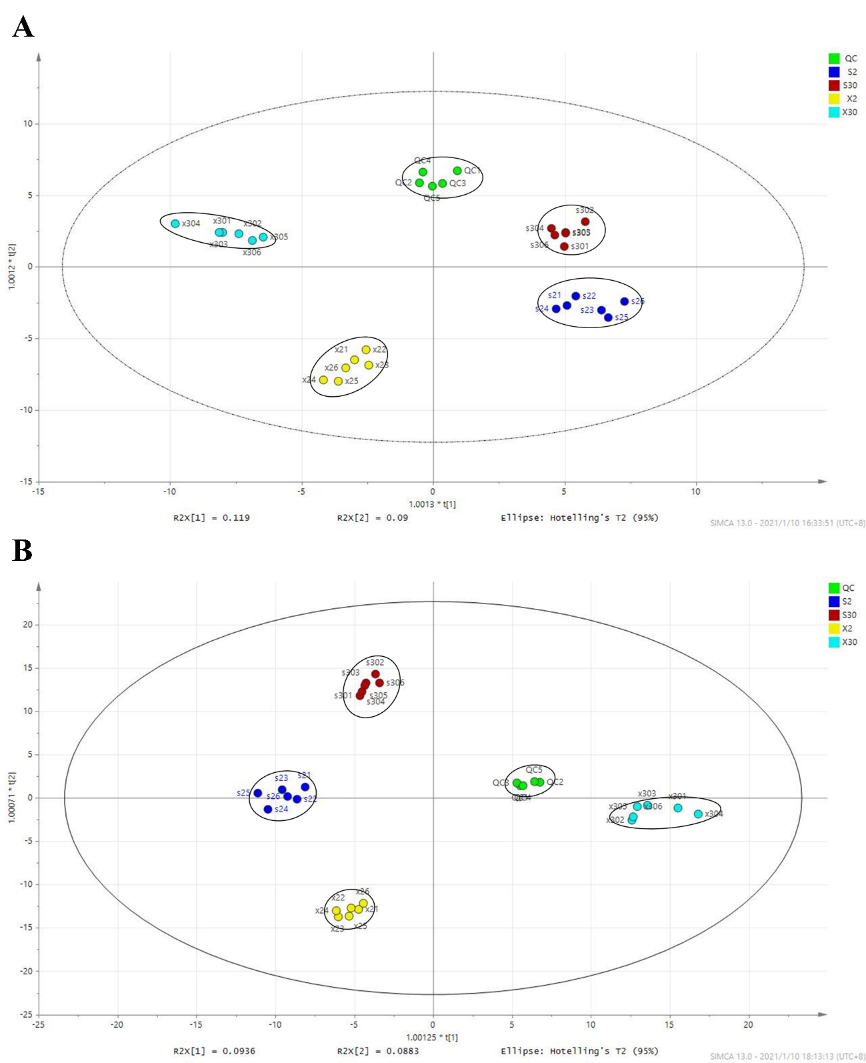


**Fig. S2. *S. cerevisiae* strain XP metabolome profiling (OPLS-DA).** (**A**) Positive ion mode. (**B**) Negative ion mode.


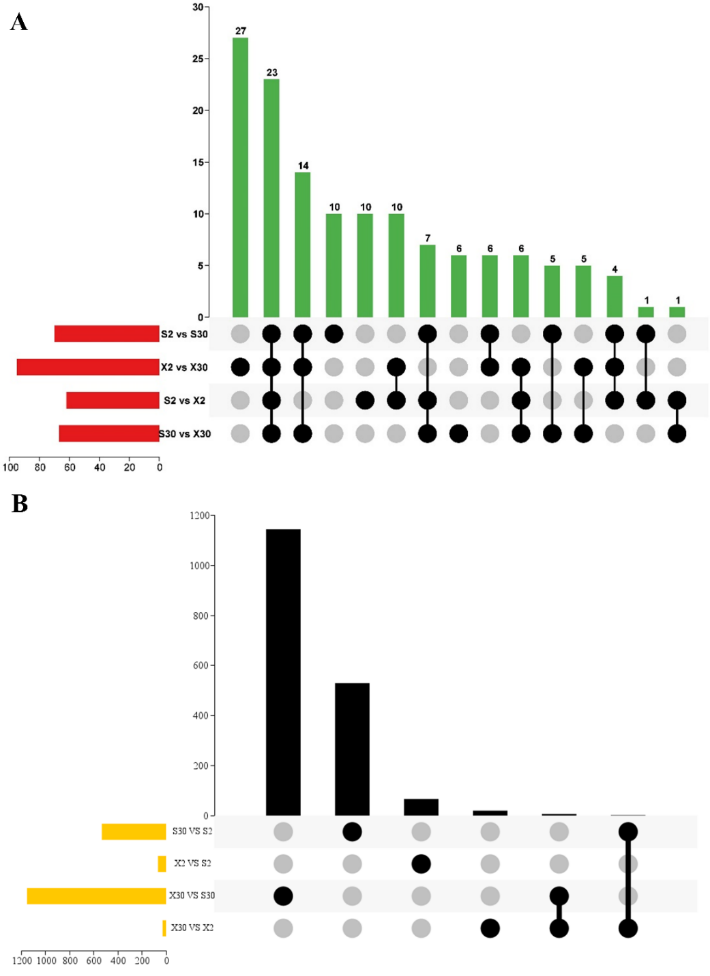


**Fig. S3. Upset plot for transcripts and metabolites.** (**A**) Metabolites. (**B**) Transcripts. The bar chart at the bottom left represents the number of metabolites/transcripts in each set, the name of each set in the middle left, the dot matrix at the bottom left corresponds to the bar chart at the top, the black dot represents the intersection of the collection lines involved, and the bar chart at the top represents the number of metabolites/transcripts corresponding to each intersection.


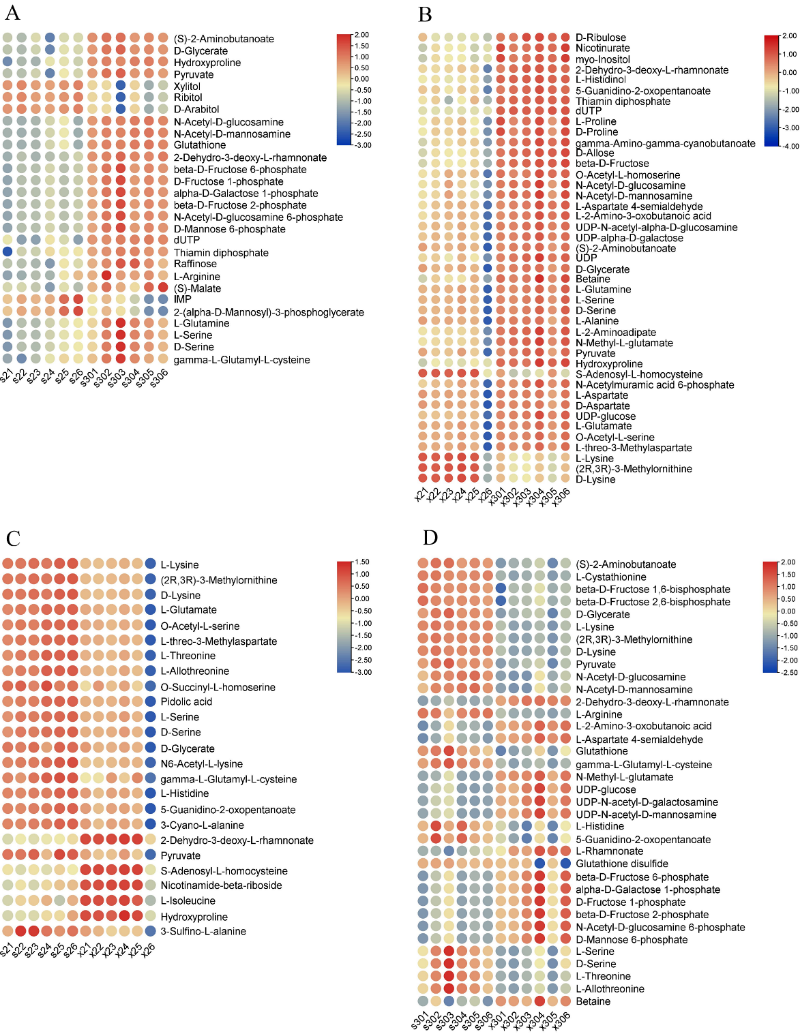


**Fig. S4.** **Comparison of different Metabolites.** Select metabolites with significant differences for analysis (p < 0.01). (**A**) S2 VS S30. (**B**) X2 VS X30. (**C**) X2 VS S2. (**D**) X30 VS S30.


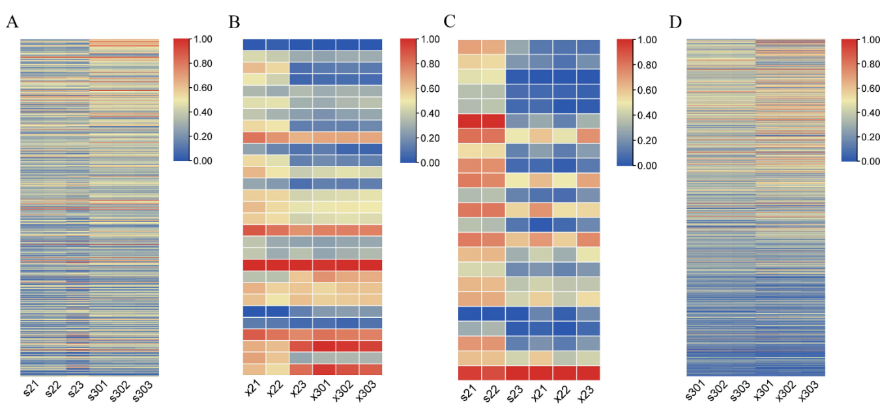


**Fig. S5. Comparison of different transcripts.** Select metabolites with significant differences for analysis (p < 0.01). (**A**) S2 VS S30. (**B**) X2 VS X30. (**C**) X2 VS S2. (**D**) X30 VS S30.

**
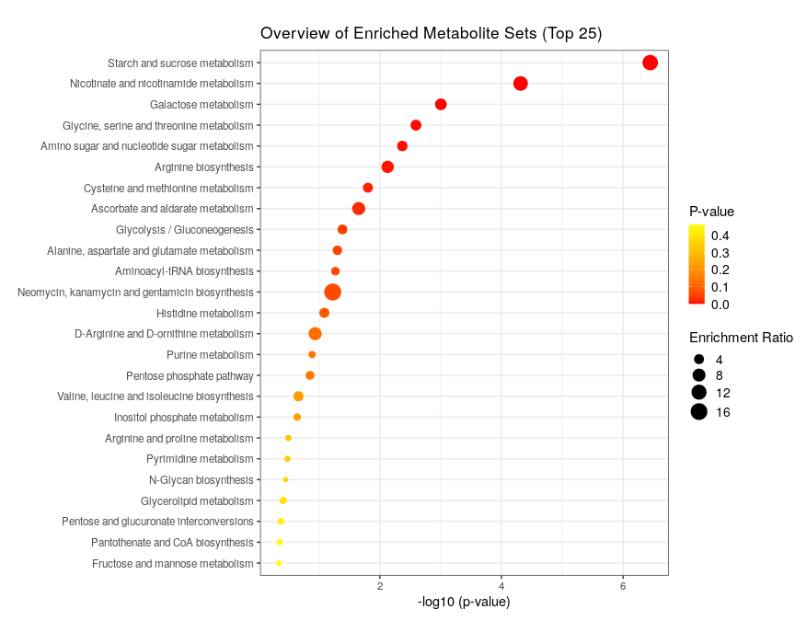
**

**Fig. S6. Metabolic pathway enrichment of X2 VS X30 vs S2 VS S30 characteristic differences.** Increase the sugar concentration from 2% to 30%, and compare the difference in metabolites between strains S288C and XP.

**
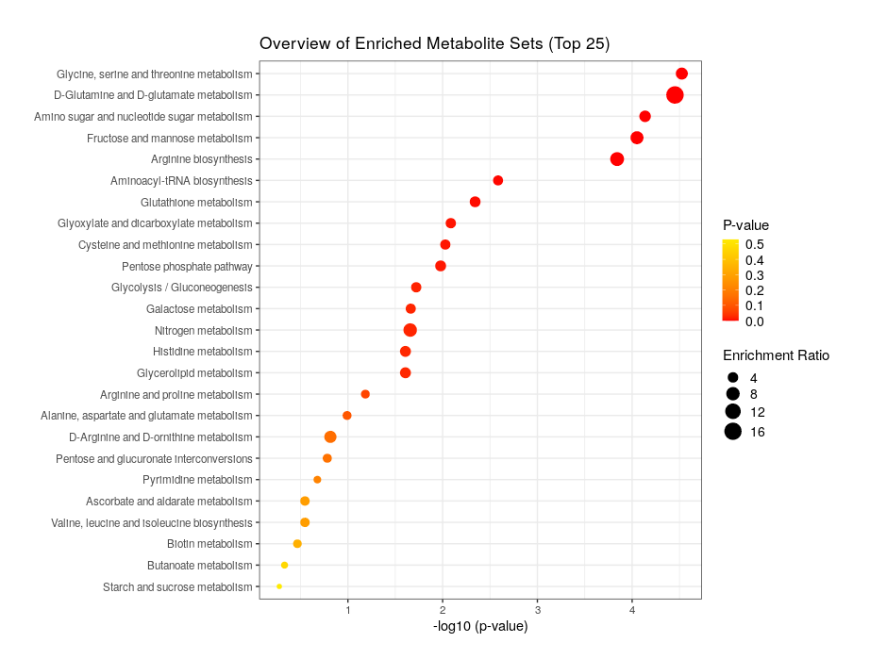
**

**Fig. S7. Metabolic pathway enrichment of characteristic differences between strains XP and S288c in the XP30 medium.** Comparison of the differences in metabolites between strains S288C and XP at 30% glucose concentration.


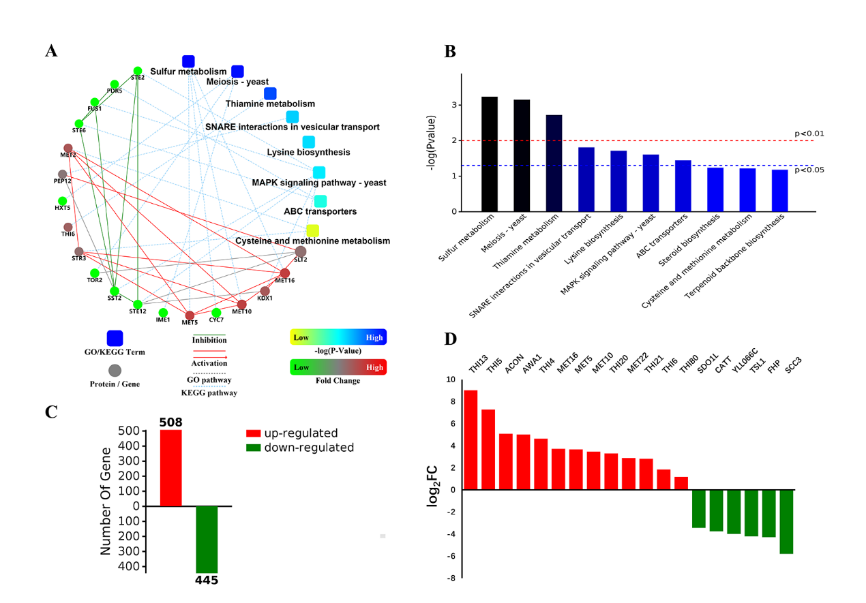


**Fig. S8. Transcriptome analysis of** ***S. cerevisiae* strain XP and *S. cerevisiae* strain S288C in the FM30 medium.** (**A**) PPI analysis of transcriptome in X30 VS S30. Circle: Transcripts; Square: Metabolic pathway. Red: Up-regulation; Green: Down-regulation; Yellow: Low p-value; Blue: High p-value. (**B**) Differential pathway between XP and S288C in FM30 medium (X30 VS S30). (**C**) The total DEGs in X30 VS S30. (**D**) The fold changes of main DEGs in X30 VS S30.


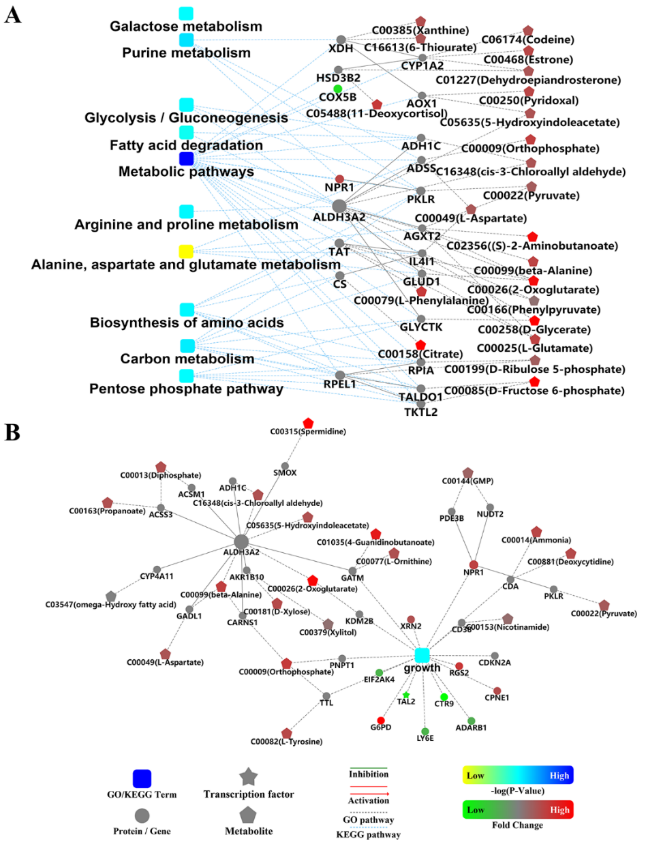


**Fig. S9. Overview of multi-omics** (**A**) PPI analysis of transcriptome and metabolome in X30 VS S30. (**B**) PPI analysis of transcriptome and metabolome about growth process in X30 VS S30. Circle: Transcripts; Pentagon: Metabolites; Star: Transcription factor; Square: Metabolic pathway. Red: Up-regulation; Green: Down-regulation. The difference is enhanced from yellow to blue. Data of transcriptome are the means from three parallel experiments. Data of metabolome are the means from six parallel experiments.


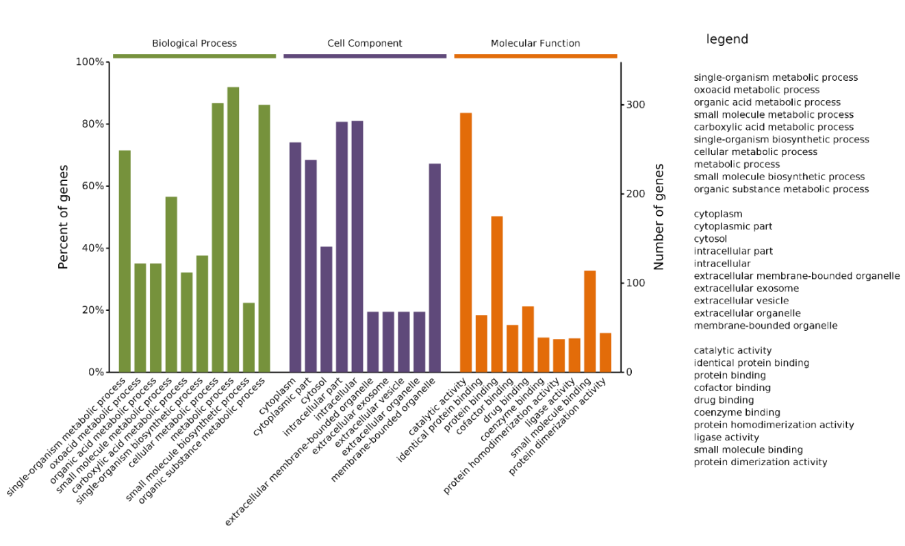


**Fig. S10. GO enrichment of differential metabolites and transcripts between X30 and S30 by Omicsbean.**


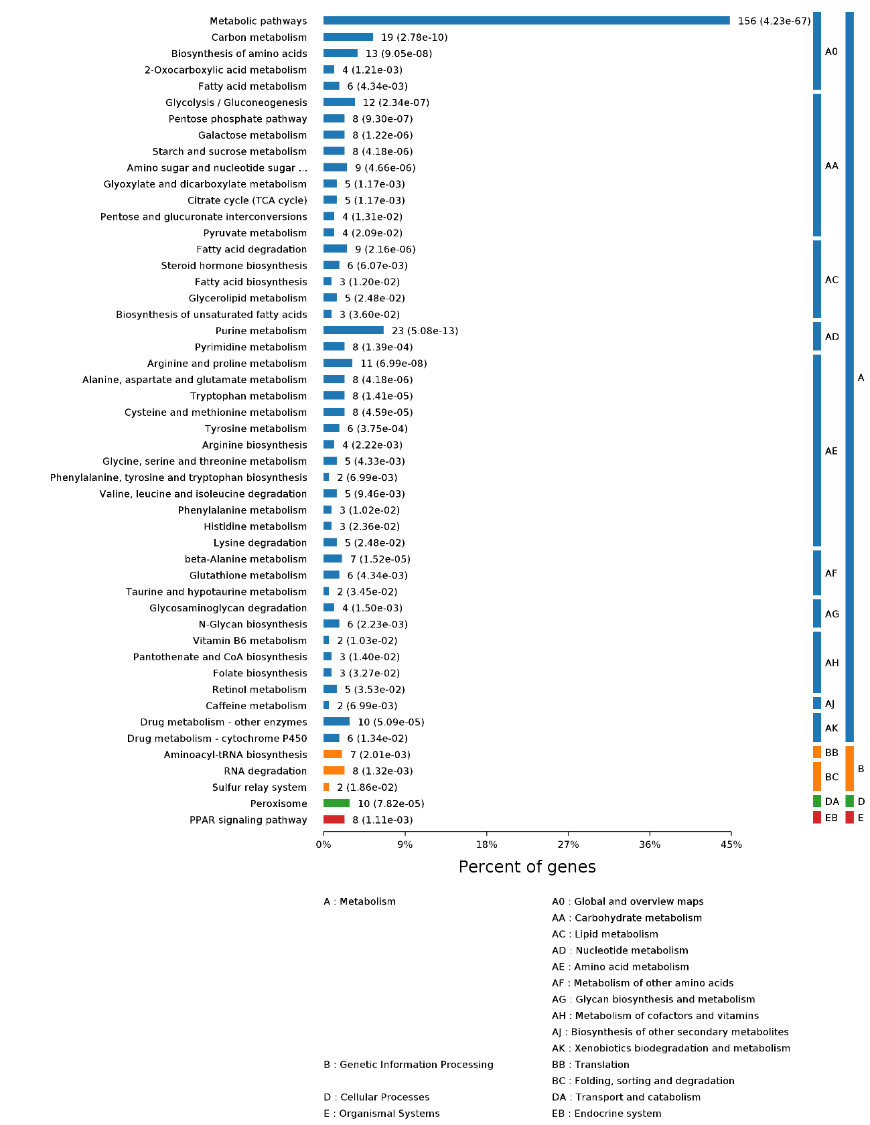


**Fig. S11. Critical differential metabolism pathway between X30 and S30 by Omicsbean.**


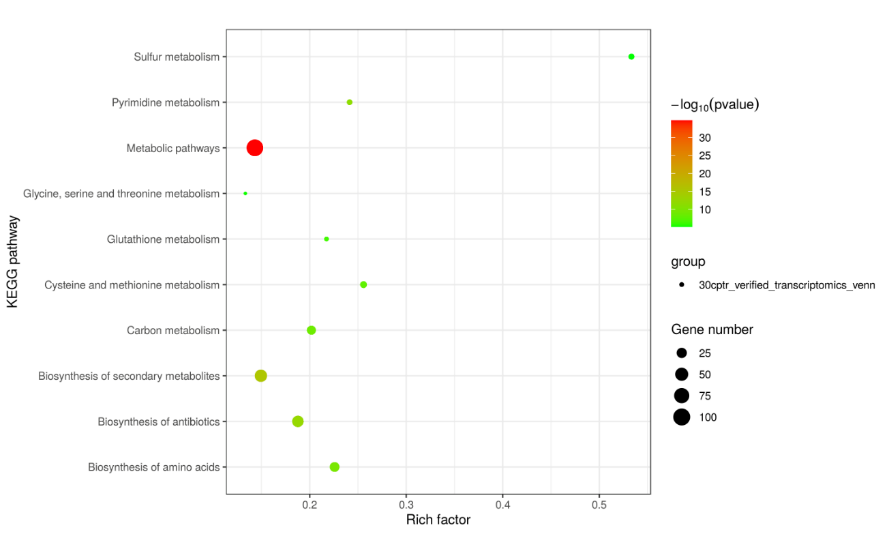


**Fig. S12. Transcription and metabolism co-enrichment bubble chart of different pathways.**


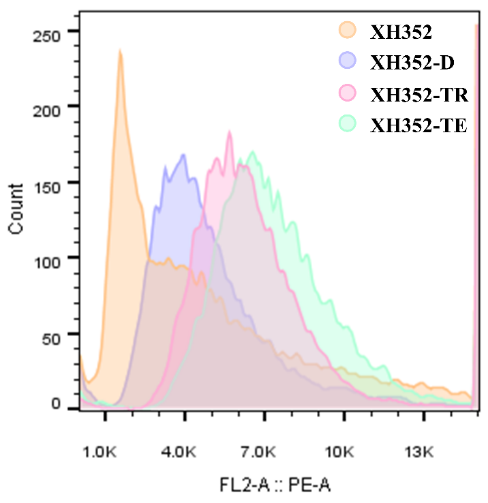


**Fig. S13. The DNA content analysis of XH352 and its polyploids.**


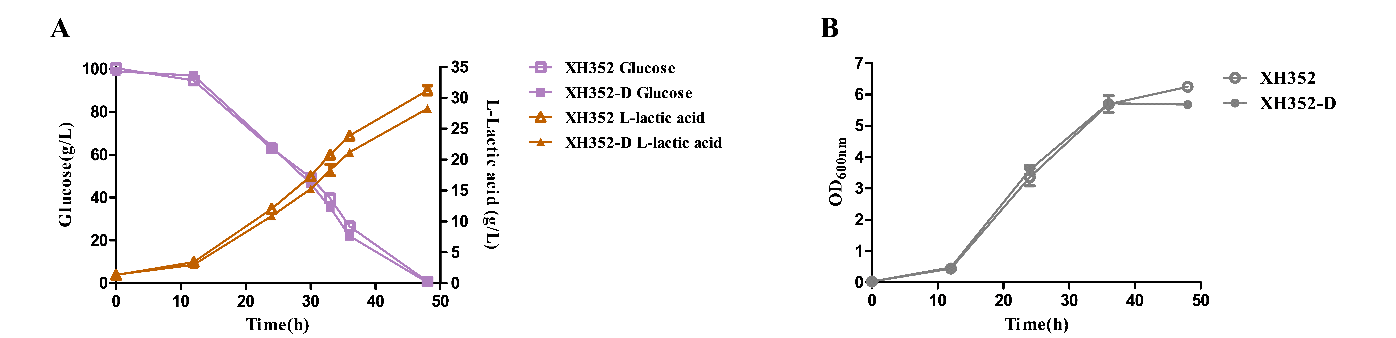


**Fig. S14. Fermentation of the haploid strain XH352 and the diploid strain XH352-D** **in a 250 mL shake flask.** (**A**) Glucose consumption and l-lactic acid production of strains XH352 and XH352-D. (**B**) Growth of strains XH352 and XH352-D.


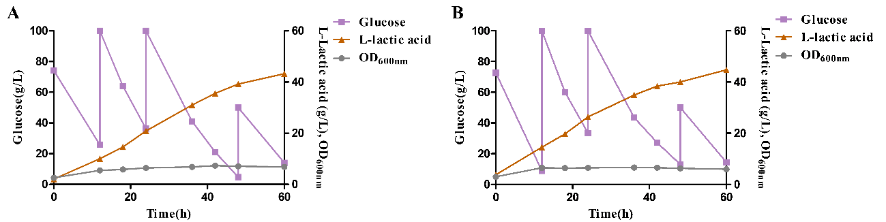


**Fig. S15. Fermentation of the polyploids.** (**A**) Fed-batch fermentation of the triploid strain XH352-TR in a 5-L fermentor. (**B**) Fed-batch fermentation of the triploid strain XH352-TE in a 5-L fermentor.


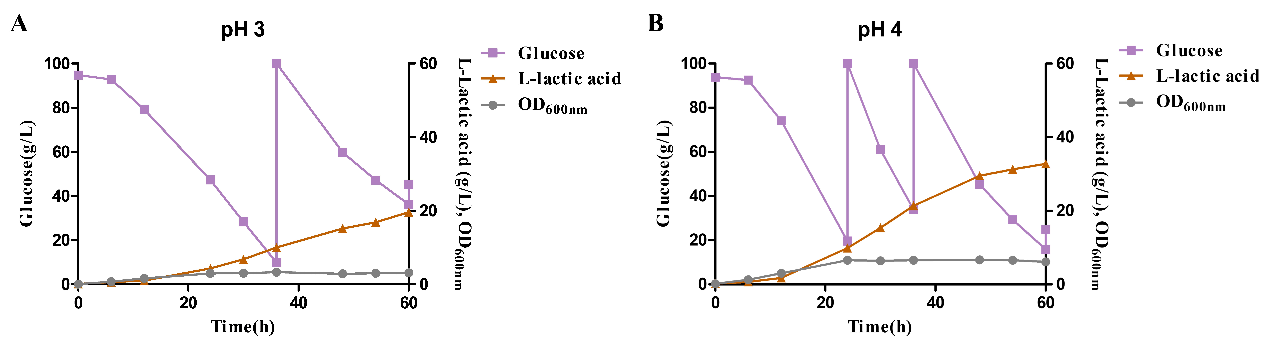


**Fig. S16. Fermentation of strain XH352-D in low pH value conditions.** (**A**) Fed-batch fermentation of the strain XH352-D in a 5-L fermentor controlled at pH 3. (**B**) Fed-batch fermentation of the strain XH352-D in a 5-L fermentor controlled at pH 4.


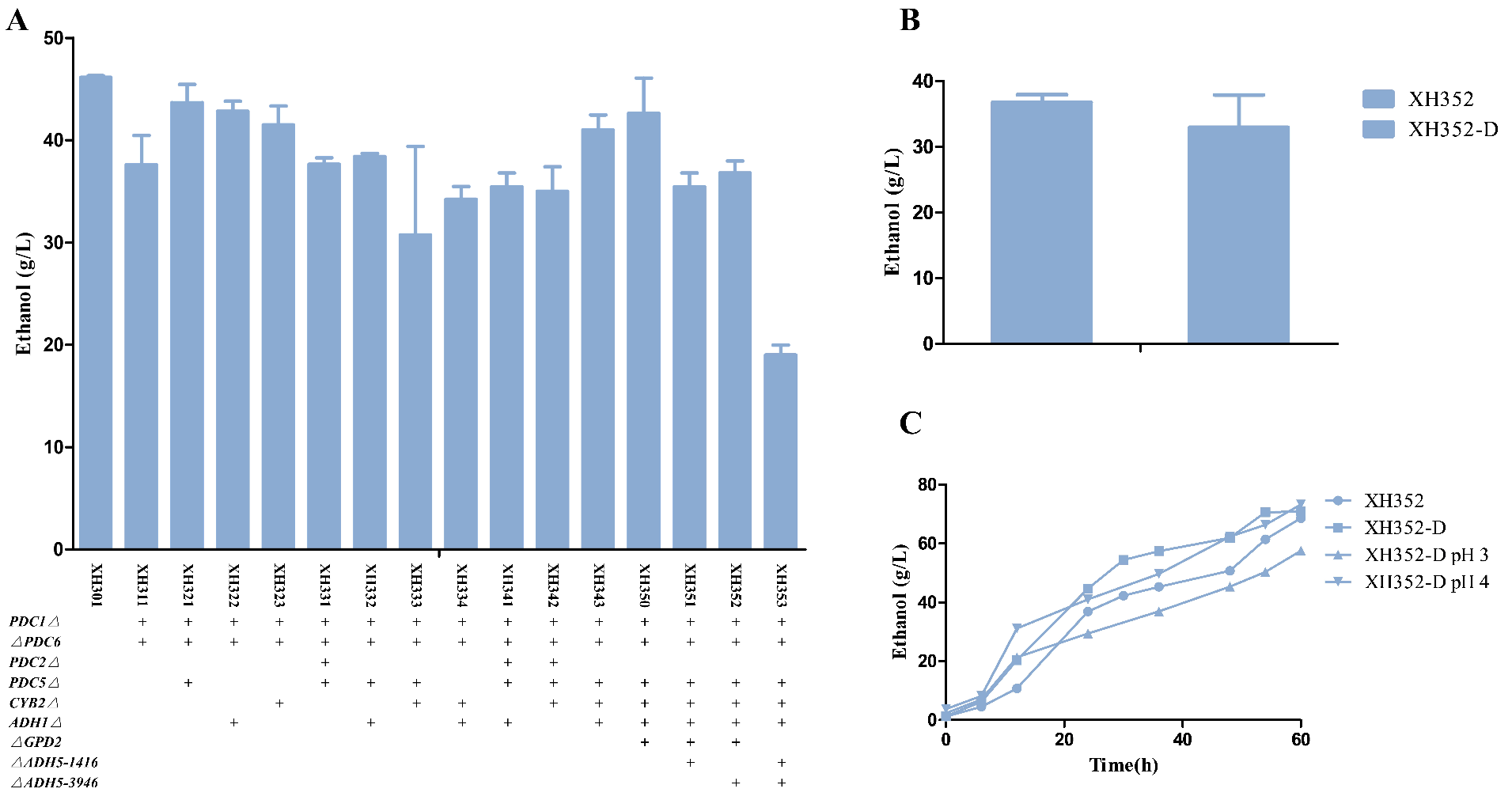


**Fig. S17. Ethanol production of the** **genetic engineering strains.** (**A**) Ethanol production in genetic engineering strains at 48h in the shake flasks. △*gene*: gene knockout by HR. *gene*△: gene blocking by NHEJ. Data are the means from three parallel experiments. Error bars indicate standard deviations from three parallel experiments. (**B**) Ethanol production of the haploid strain XH352 and the diploid strain XH352-D at 48h in the shake flasks. Data are the means from three parallel experiments. Error bars indicate standard deviations from three parallel experiments. (**C**) Ethanol production of XH352 and its polyploid strains in the 5-L fermenter. The pH values were controlled at 5, 4 and 3. Data are the means from three parallel experiments. Error bars indicate standard deviations from three parallel experiments.


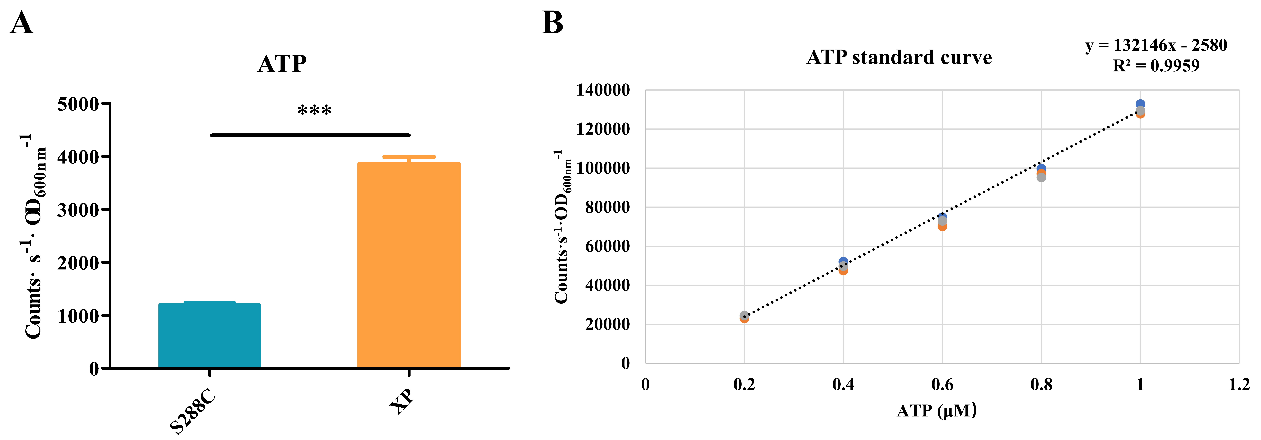


Fig. S18. Determination of the ATP levels. (A) The ATP levels of Strain XP and S288C in the FM30 medium. Data are the means from three parallel experiments. Error bars indicate standard deviations from three parallel experiments. *** P < 0.001. (B) The standard curve of the ATP levels. The formula for the linear trend line was derived from the least square fit. There are three biological replicates per concentration.

**Supplementary Tables**

**Table S1** Strains used in this study.

| Strain | Reference or source | species | Relevant characteristic(s) |
| --- | --- | --- | --- |
| XP | This study | *Saccharomyces cerevisiae* | Wild type |
| S288C | GCF_000146045.2 | *Saccharomyces cerevisiae* | Standard |
| BY4741 | GCA_000766575.2 | *Saccharomyces cerevisiae* | Standard |
| Ethanol Red | GCA_001078105.1 | *Saccharomyces cerevisiae* | Industry standard |
| XP-3 | This study | *Saccharomyces cerevisiae* | XP△*Ura3* |
| XP-H | This study | *Saccharomyces cerevisiae* | Haploid of XP |
| XP-H3 | This study | *Saccharomyces cerevisiae* | Haploid of XP-3 |
| XP-H-D | This study | *Saccharomyces cerevisiae* | Diploid of XP-H by mating |
| XH301 | This study | *Saccharomyces cerevisiae* | XP-H3 expressing *BcLDH_H-1_* from *Bacillus coagulans* H-1 |
| XH302 | This study | *Saccharomyces cerevisiae* | XP-H3 expressing *BcLDH_2-6_* from *Bacillus coagulans* 2-6 |
| XH303 | This study | *Saccharomyces cerevisiae* | XP-H3 expressing *BtLDH* from *Bos taurus* |
| XH304 | This study | *Saccharomyces cerevisiae* | XP-H3 expressing *LdLDH* from *Lactobacillus helveticus* |
| XH305 | This study | *Saccharomyces cerevisiae* | XP-H3 expressing *LmLDH* from *Leuconostoc mesenteroides* |
| XH306 | This study | *Saccharomyces cerevisiae* | XP-H3 expressing *RoLDH* from *Rhizopus oryzae* |
| XH310 | This study | *Saccharomyces cerevisiae* | XH301△*PDC6* |
| XH311 | This study | *Saccharomyces cerevisiae* | XH310*PDC1*△ |
| XH321 | This study | *Saccharomyces cerevisiae* | XH311*PDC5*△ |
| XH322 | This study | *Saccharomyces cerevisiae* | XH311*PDC2*△ |
| XH323 | This study | *Saccharomyces cerevisiae* | XH311*ADH1*△ |
| XH324 | This study | *Saccharomyces cerevisiae* | XH311*CYB2*△ |
| XH331 | This study | *Saccharomyces cerevisiae* | XH311*PDC2*△*PDC5*△ |
| XH332 | This study | *Saccharomyces cerevisiae* | XH311*ADH1*△*PDC5*△ |
| XH333 | This study | *Saccharomyces cerevisiae* | XH311*CYB2*△*PDC5*△ |
| XH334 | This study | *Saccharomyces cerevisiae* | XH311*ADH1*△*CYB2*△ |
| XH341 | This study | *Saccharomyces cerevisiae* | XH311*PDC2*△*PDC5*△*ADH1*△ |
| XH342 | This study | *Saccharomyces cerevisiae* | XH311*PDC2*△*PDC5*△*CYB2*△ |
| XH343 | This study | *Saccharomyces cerevisiae* | XH311*PDC5*△*ADH1*△*CYB2*△ |
| XH350 | This study | *Saccharomyces cerevisiae* | XH343△*GPD2* |
| XH351 | This study | *Saccharomyces cerevisiae* | XH350△*ADH5-1416* |
| XH352 | This study | *Saccharomyces cerevisiae* | XH350△*ADH5-3946* |
| XH353 | This study | *Saccharomyces cerevisiae* | XH351△*ADH5-3946* |
| XH352-D | This study | *Saccharomyces cerevisiae* | Diploid of XH352 |
| XH352-TR | This study | *Saccharomyces cerevisiae* | Triploid of XH352 |
| XH352-TE | This study | *Saccharomyces cerevisiae* | Tetraploid of XH352 |
| Top10 | This study | *Escherichia coli* | Plasmid cloning |

**Table S2** plasmids used in this study.

| Plasmid | Reference or source | Relevant characteristic(s) |
| --- | --- | --- |
| pRS416 | Prof. Xie provided | Ap^r^, URA^+^, expressing vector, carries *URA3* auxotrophic marker |
| pUG6 | Prof. Hou provided | Ap^r^, Kan^r^, carries G418 resistance marker with loxP sites on both sides |
| pML104 | Addgene | Ap^r^, URA^+^, gene editing vector, carries *Cas9* from *Streptococcus pyogenes* and *C*-terminal portion of gRNA scaffold |
| p414-TEF1p-Cas9-CYC1t | Prof. Hou provided | Ap^r^, NAT1^r^, gene editing vector, carries *Cas9* from *Streptococcus pyogenes* |
| pRS42H | Prof. Hou provided | Ap^r^, HyB^r^, gene editing vector, carries gRNA scaffold |
| pSH65 | Addgene | Ap^r^, Ble^r^, gene knockout vector, carries *Cre* recombinase coding gene |
| pRS416-*P*_adh1_-HOM2 | This study | Ap^r^, URA^+^, expressing homothallic switching endonuclease HO |
| pRS416-*P*_adh1_-*BcLDH_H-1_* | This study | Ap^r^, URA^+^, gene expression vector, carrying lactic dehydrogenase from *Bacillus coagulans* H-1 |
| pRS416-*P*_adh1_-*Bcldh_2-6_* | This study | Ap^r^, URA^+^, gene expression vector, carrying lactic dehydrogenase from *Bacillus coagulans* 2-6 |
| pRS416-*P*_adh1_-*BtLDH* | This study | Ap^r^, URA^+^, gene expression vector, carrying lactic dehydrogenase from *Bos taurus* |
| pRS416-*P*_adh1_-*LdLDH* | This study | Ap^r^, URA^+^, gene expression vector, carrying lactic dehydrogenase from *Lactobacillus helveticus* |
| pRS416-*P*_adh1_-*LmLDH* | This study | Ap^r^, URA^+^, gene expression vector, carrying lactic dehydrogenase from *Leuconostoc mesenteroides* |
| pRS416-*P*_adh1_-*RoLDH* | This study | Ap^r^, URA^+^, gene expression vector, carrying lactic dehydrogenase from *Rhizopus oryzae* |

**Table S3** Sequences of primers used in this study.

| Primer | Sequence (5’-3’) |
| --- | --- |
| *URA3*KOup-F | ATGTCGAAAGCTACATATAA |
| *URA3*KOup-RV | CATACATTATACGAAGTTATTCAGTCAAGATATCCACATG |
| *URA3*-*KanMX*-F1 | CATGTGGATATCTTGACTGAATAACTTCGTATAATGTATG |
| *URA3*-*KanMX*-RV1 | CCACCCATGTCTCTTTGAGCATAACTTCGTATAGCATACAT |
| *URA3*KOdown-F1 | ATGTATGCTATACGAAGTTATGCTCAAAGAGACATGGGTGG |
| *URA3*KOdown-RV1 | TTAGTTTTGCTGGCCGCATC |
| *URA3*up-RV2 | AGGGTTGTCGACCTGCAGTGTGCCCTCCATGGAAAAAT |
| *URA3*KOdown-F2 | GTGATATCAGATCCACTAGTGGCGTGTATACAGAATAGCAGAATGGGC |
| *URA3*KOdown-RV2 | ACCCTTAGTATATTCTCCAGTAGC |
| *URA3*-*KanMX*-RV2 | GCCCATTCTGCTATTCTGTATACACGCCACTAGTGGATCTGATATCAC |
| *URA3*-*KanMX*-F2 | ATTTTTCCATGGAGGGCACAGCTGCAGGTCGACAACCCT |
| XP*PDC1*sgRNA-F | GATCGGAAGTCATTGACACCATCT GTTTTAGAGCTAG |
| XP*PDC1*sgRNA-RV | CTAGCTCTAAAAC AGATGGTGTCAATGACTTCC |
| P1 | AGTCACATCAAGATCGTTTATGG |
| P2 | GCACGGAATATGGGACTACTTCG |
| P3 | ACTCCACTTCAAGTAAGAGTTTG |
| HO19-F | GCGAATTCATGCTTTCTGAAAACACG |
| HO19-R | ACAAGCATGCGTCTTCTCGTTAAGACTG |
| HOM1-F | TGACGGTACAACAAAAGAGC |
| HOM1-R | GTACCGTCACCTAACCACAGACC |
| HOM2-F | CAGCATAAAATATCACAAAA |
| HOM2-R | TTTATGCTGTTCTCCCACAC |
| 416-HOM2-F | CAACTGGATCC ATGCTTTCTGAAAACACG |
| 416-HOM2-R | TTCGCGAATTC TTAGCAGATGCGCGCA |
| 416-*BcLDH_H-1_*-F | GCATACAATCAACTGGATCC ATGAAAAAAGTAAACCGTGT |
| 416-*BcLDH_H-1_*-RV | CATAAGAAATTCGCGAATTC TTACAATATCGGTGCCATTG |
| 416-*BcLDH_2-6_*-F | GCATACAATCAACTGGATCC ATGAAAAAGGTCAATCGTAT |
| 416-*BcLDH_2-6_*-RV | CATAAGAAATTCGCGAATTC TTACAATACAGGTGCCATCG |
| 416-*BtLDH*-F | AGCTATACCAAGCATACAATCAACT GGATCC ATGGCCACCCTGAAGGAC |
| 416-*BtLDH*-RV | AAAATCATAAATCATAAGAAATTCGC GAATTC TTAGAACTGCAGCTCCTTCTG |
| 416-*LdLDH*-F | AGCTATACCAAGCATACAATCAACT GGATCC ATGGCCCGAGAGGAGAA |
| 416-*LdLDH*-RV | AAAATCATAAATCATAAGAAATTCGC GAATTC TTACTGTCGCACCTTGACG |
| 416-*LmLDH*-F | AGCTATACCAAGCATACAATCAACT GGATCC ATGAAGATTTTTGCTTACGGCAT |
| 416-*LmLDH*-RV | AAAATCATAAATCATAAGAAATTCGC GAATTC TTAATATTCAACAGCAATAGCTGGC |
| 416-*RoLDH*-F | ATCGGGATCC ATGATTCCTATGGTACTGCACTCAAAGG |
| 416-*RoLDH*-RV | ATCGGAATTC TCAAAACATGATTTATTATTTGTAA |
| *PDC6*-UP-F | TTATAAGACAAGCGCAGGGCCA |
| *PDC6*-UP-RV | AGGGTTGTCGACCTGCAGC GCCATTAGTAGTGTACTCAAAAACGA |
| *PDC6*-G418-F | TCGTTTTTGAGTACACTACTAATGGC GCTGCAGGTCGACAACCCT |
| *PDC6*-G418-RV | TATAGCAAAAACATATTGCCAACAAA GCCACTAGTGGATCTGATATCAC |
| *PDC6*-DOWN-F | GTGATATCAGATCCACTAGTGGC TTTGTTGGCAATATGTTTTTGCTATA |
| *PDC6*-DOWN-RV | CACGTTCCCTTTTCATAAAACAC |
| *CYB2*-sgRNA1-pRS42H-F | GATCACAACCCAACAATCATGTTTTAGAGCTAGAAATAGCAAG |
| *CYB2*-sgRNA1-pRS42H-RV | ATGATTGTTGGGTTGTGATCGATCATTTATCTTTCACTGCGGA |
| *CYB2*-sgRNA2-pRS42H-F | GCTGTAGCAGACACGTAGAA GTTTTAGAGCTAGAAATAGCAAG |
| *CYB2*-sgRNA2-pRS42H-RV | TTCTACGTGTCTGCTACAGCGATCATTTATCTTTCACTGCGGA |
| *PDC2*-sgRNA1-pRS42H-F | TGGCGGAGAGGCACCCAAAG GTTTTAGAGCTAGAAATAGCAAG |
| *PDC2*-sgRNA1-pRS42H-RV | CTTTGGGTGCCTCTCCGCCA GATCATTTATCTTTCACTGCGGA |
| *PDC2*-sgRNA2-pRS42H-F | AAAGTGGACGCAACTTGAAT GTTTTAGAGCTAGAAATAGCAAG |
| *PDC2*-sgRNA2-pRS42H-RV | ATTCAAGTTGCGTCCACTTT GATCATTTATCTTTCACTGCGGA |
| *PDC5*-sgRNA1-pRS42H-F | GACTGGTCTTTGGGTAGTGT GTTTTAGAGCTAGAAATAGCAAG |
| *PDC5*-sgRNA1-pRS42H-RV | ACACTACCCAAAGACCAGTC GATCATTTATCTTTCACTGCGGA |
| *PDC5*-sgRNA2-pRS42H-F | AACTGTTGTTGAATTGATCA GTTTTAGAGCTAGAAATAGCAAG |
| *PDC5*-sgRNA2-pRS42H-RV | TGATCAATTCAACAACAGTT GATCATTTATCTTTCACTGCGGA |
| XP*ADH1*sgRNA1-F | GATC TCTATCCCAGAAACTCAAAA GTTTTAGAGCTAG |
| XP*ADH1*sgRNA1-R | CTAGCTCTAAAAC TTTTGAGTTTCTGGGATAGA |
| XP*ADH1*sgRNA2-F | GATC CTTTGTATTCCAACTTACCG GTTTTAGAGCTAG |
| XP*ADH1*sgRNA2-R | CTAGCTCTAAAAC CGGTAAGTTGGAATACAAAG |
| XP*GPD2*sgRNA1-pRS42H-F | GCACGACGAGTATATAACAC GTTTTAGAGCTAGAAATAGCAAG |
| XP*GPD2*sgRNA1-pRS42H-R | GTGTTATATACTCGTCGTGC GATCATTTATCTTTCACTGCGGA |
| XP*GPD2*sgRNA2-pRS42H-F | CGCAATGACTTTGGCGATGG GTTTTAGAGCTAGAAATAGCAAG |
| XP*GPD2*sgRNA2-pRS42H-R | CCATCGCCAAAGTCATTGCG GATCATTTATCTTTCACTGCGGA |
| XP*ADH5-3946*-sgRNA1-pRS42H-F | ATGGGCAGCTTGAACGGCA GTTTTAGAGCTAGAAATAGCAAG |
| XP*ADH5-3946*-sgRNA1-pRS42H-R | TGCCGTTCAAGCTGCCCAT GATCATTTATCTTTCACTGCGGA |
| XP*ADH5-1416*-sgRNA1-pRS42H-F | ACAGCAGCAATGCACTTAAT GTTTTAGAGCTAGAAATAGCAAG |
| XP*ADH5-1416*-sgRNA1-pRS42H-R | ATTAAGTGCATTGCTGCTGT GATCATTTATCTTTCACTGCGGA |
| *ADH5-1416*-F | ATGTCCGCCGCTACTGTT |
| *ADH5-1416*-donor-up-RV | AGCAGAGCCTTTCCACAC TACGATACCGGCTCCTTCGT |
| *ADH5-1416*-donor-down-F | ACGAAGGAGCCGGTATCGTA GTGTGGAAAGGCTCTGCT |
| *ADH5-1416*-RV | CATCAGACTTCAAGACGGTTC |
| *ADH5-3946*-F | ATGCCTTCGCAAGTCATTCC |
| *ADH5-3946*-donor-up-RV | ACAACTTGATTGAAAACATCGGA TTCGTGACCACCGATTAATG |
| *ADH5-3946*-donor-down-F | CATTAATCGGTGGTCACGAA TCCGATGTTTTCAATCAAGTTGT |
| *ADH5-3946*-RV | GAAGTCTCAACAACATATCTACC |
| *GPD2*-F | ATGCTTGCTGTCAGAAGATT |
| *GPD2*-donor-up-RV | TTACCGGTCTTGGCCATGTAT ACACGGCAGAGTCCGATCTT |
| *GPD2*-donor-down-F | AAGATCGGACTCTGCCGTGT ATACATGGCCAAGACCGGTAA |
| *GPD2*-RV | CATCGATGTCTAGCTCTTCAATC |

**Table S4. The P value of two-tailed T-test for the doubling times.**

| Wild Type Strain | Standard Strain | Medium | | | |
| --- | --- | --- | --- | --- | --- |
|  |  | YPD2 | YPD30 | FM | FM30 |
| XP | S288C | < 0.0001*** | < 0.0001*** | 0.0136* | < 0.0001*** |
|  | Ethanol Red | 0.0003*** | 0.0019** | 0.0025** | < 0.0001*** |
|  | BY4741 | 0.0027** | < 0.0001*** | 0.019* | < 0.0001*** |

*P < 0.05, **P <0.01, ***P < 0.001.

**Table S5** Transcriptome DEGs function in this study (X30 VS S30).

|  | DEGs | log2FC | Effect^a^ |
| --- | --- | --- | --- |
| Thiamine biosynthesis | *THI13* | 9.02196 | Responsible for the formation of the pyrimidine heterocycle in the thiamine biosynthesis pathway. Catalyzes the formation of hydroxymethylpyrimidine phosphate (HMP-P) from histidine and pyridoxal phosphate (PLP). |
|  | *THI4* | 4.63624 | Involved in biosynthesis of the thiamine precursor thiazole. Catalyzes the conversion of NAD and glycine to adenosine diphosphate 5-(2-hydroxyethyl)-4-methylthiazole-2-carboxylic acid (ADT). |
|  | *THI20* | 3.30527 | Catalyzes the phosphorylation of hydroxymethylpyrimidine phosphate (HMP-P) to HMP-PP, and also probably that of HMP to HMP-P. |
|  | *THI5* | 7.27096 | Responsible for the formation of the pyrimidine heterocycle in the thiamine biosynthesis pathway. Catalyzes the formation of hydroxymethylpyrimidine phosphate (HMP-P) from histidine and pyridoxal phosphate (PLP). |
| Mitochondrial protection | *ACON* | 5.0841 | Plays also an essential role in mtDNA maintenance. May directly protect mtDNA from accumulation of point mutations and ssDNA breaks as a component of mitochondrial nucleoids, or by preventing accumulation of iron citrate thereby alleviating its detrimental effects in mitochondria. |
| Sulfur metabolism | *MET16* | 3.72437 | The NADP dependent reduction of PAPS into sulfite involves thioredoxin which probably plays the role of a thiol carrier. |
|  | *MET5* | 3.6642 | Catalyzes the reduction of sulfite to sulfide. |
|  | *MET10* | 3.47069 | This enzyme catalyzes the 6-electron reduction of sulfite to sulfide. |
|  | *MET22* | 2.8826 | Converts adenosine 3'-phosphate 5'-phosphosulfate (PAPS) to adenosine 5'-phosphosulfate (APS) and 3'(2')-phosphoadenosine 5'- phosphate (PAP) to AMP. |
|  |  |  | Salt tolerance. Confers resistance to lithium. |
|  | *THI21* | 2.82404 | Catalyzes the phosphorylation of hydroxymethylpyrimidine phosphate (HMP-P) to HMP-PP, and also probably that of HMP to HMP-P. |
|  | *THI6* | 1.84941 | Condenses 4-methyl-5-(beta-hydroxyethyl)thiazole monophosphate (THZ-P) and 2-methyl-4-amino-5-hydroxymethyl pyrimidine pyrophosphate (HMP-PP) to form thiamine monophosphate (TMP) |
|  | *THI80* | 1.16843 | The only enzyme in yeast capable of synthesizing thiamine pyrophosphate (TPP). |
| Robustness | *CATT* | -3.7506 | Occurs in almost all aerobically respiring organisms and serves to protect cells from the toxic effects of hydrogen peroxide. |
|  | *AWA1* | 5.01945 | Involved in cell wall organization and biosynthesis. Confers cell surface hydrophobicity (CSH). |
|  | *FHP* | -4.2911 | Involved in NO detoxification in an aerobic process. Protects the fungus from various noxious nitrogen compounds. |
|  | *TSL1* | -4.205 | Catalyzes the production of trehalose from glucose-6-phosphate and UDP-glucose. |
|  |  |  | Confers sensitivity to physiological concentrations of phosphate and to fructose 6-phosphate. |
| Cell cycle | *YLL066C* | -3.9916 | Catalyzes DNA unwinding and is involved in telomerase-independent telomere maintenance. |
|  | *SCC3* | -5.7947 | Codes a complex required for the cohesion of sister chromatids after DNA replication |
|  | *SDO1L* | -3.4369 | Influences telomere capping. |

^a^ Functional comments are from the Uniprot database.

**Table S6** The function of important nodes in PPI analysis (X30 VS S30).

| Gene | Effect ^a^ |
| --- | --- |
| *TAL2* | Balances metabolites in the pentose-phosphate pathway. |
| *ALDH3A2* | Catalyzes the oxidation of medium and long-chain aliphatic aldehydes to fatty acids with NADH production. |
| *CTR9* | RNA polymerase-associated protein. Involved in transcription initiation via genetic interactions with TATA-binding proteins. |
| *LY6E* | Relieved the inhibitory effect of *EIF2AK2/PKR* on cell proliferation and migration while reduced exocytosis and maintaining membrane stability. |
| *NTR1* | A nutrient-regulated protein kinase up-regulated, promoting the nitrogen-containing nutrient transport system. |

^a^Functional comments are from the Uniprot database.
